# Supplementary material for: Wolfberry genomes and the evolution of Lycium (Solanaceae)
Source: Commun Biol. 2021 Jun 3;4:671. doi: 10.1038/s42003-021-02152-8 (PMC8175696; doi:10.1038/s42003-021-02152-8)
Supplement: Supplementary file 2 — Supplementary Information [file 42003_2021_2152_MOESM2_ESM.pdf]

Supplementary Information

Wolfberry genomes and the evolution of *Lycium*

You-Long Cao[1,2*†], Yan-long Li[1,2*], Yun-Fang Fan[1,2*], Zhen Li[3,4*], Kouki Yoshida[5*], Jie-Yu Wang[6,7], Xiao-Kai Ma[6], Ning Wang[8], Nobutaka Mitsuda[9], Toshihisa Kotake[10], Takeshi Ishimizu[11], Kun-Chan Tsai[12], Shan-Ce Niu[13], Diyang Zhang[6,14], Wei-Hong Sun[6,14], Qing Luo[1,2], Jian-Hua Zhao[1,2], Yue Yin[1,2], Bo Zhang[1,2], Jun-Yi Wang[1,2], Ken Qin[1,2], Wei An[1,2], Jun He[1,2], Guo-Li Dai[1,2], Ya-Jun Wang[1,2], Zhi-Gang Shi[1,2], En-Ning Jiao[1,2], Peng-Ju Wu[1,2], Xuedie Liu[6,14], Bin Liu[6,14], Xing-Yu Liao[6,14], Yu-Ting Jiang[6,14], Xia Yu[6,14], Yang Hao[6,14], Xin-Yu Xu[6,14], Shuang-Quan Zou[6,14], Ming-He Li[6], Yu-Yun Hsiao[15], Yu-Fu Lin[16], Chieh-Kai Liang[17], You-Yi Chen[17], Wan-Lin Wu[16], Hsiang-Chai Lu[6], Si-Ren Lan[6,14], Zhi-Wen Wang[18], Xiang Zhao[18], Wen-Ying Zhong[18], Chuan-Ming Yeh[9,11,19†], Wen-Chieh Tsai[15,16,17†], Yves Van de Peer[3,4,20,21†], Zhong-Jian Liu[1,2,6,14†]

Content

Supplementary Notes **1**

Supplementary Note 1. Preparation of haploid sequencing samples 1

Supplementary Note 2. Gene family identification and phylogenomic dating 1

Supplementary Figures **3**

Supplementary Figure 1. Cytogenetic analysis and comparison of haploid and diploid plants of *L. barbarum*.. 3

Supplementary Figure 2. Intensity signal heat map of Hi-C chromosome interaction of *L. barbarum* genome 4

Supplementary Figure 3. GO secondary functional enrichment for the expanded gene families in *L. barbarum*. 5

Supplementary Figure 4. KEGG pathway functional enrichment for the expanded gene families in *L. barbarum*. 6

Supplementary Figure 5. Phylogenetic tree of genes involved in triose phosphate/phosphate translocator (TPT) in Solanaceae. The motif M10-M13-M15-M9 presents a variation in *Lycium* species. 7

Supplementary Figure 6. Phylogenetic tree of genes involved in zeaxanthin epoxidase and xanthoxin oxidase in Solanaceae. 8

Supplementary Figure 7. K_S age distributions for the whole paranome (grey) and anchor pairs (green) in *L. barbarum* and *L. ruthenicum*.. 9

Supplementary Figure 8. K_S age distributions for the whole paranome (grey) and anchor pairs (green) in several other species from Solanaceae and Convolvulaceae and *C. canephora* 10

Supplementary Figure 9. Dot plot for inter-genomic comparison between *L. barbarum* and *V. vinifera*.. 11

Supplementary Figure 10. Distributions of synonymous substitutions per synonymous site (K_S) of one-to-one orthologs identified between species from Solanaceae and Convolvulaceae and *C. canephora*. 12

Supplementary Figure 11. Maximum likelihood phylogenetic tree of the RNase-T2-like genes obtained from wolfberry and S-RNase-related RNase-T2s obtained from species possessing S-RNase-based amino acids in Solanaceae. 15

Supplementary Figure 12. Maximum likelihood phylogenetic tree of the RNase-T2 genes obtained from *L. barbarum* and S-RNase genes from *Lycium* species cited by Miller *et al*.[5]. The tree was constructed in PhyML 3.0. Red color showed SI candidate *Lba02g01102* gene. 16

Supplementary Figure 13. YF-box genes distribution along chromosome 2 of *L. barbarum*. Green color, SI candidate *Lba02g01102* gene; red color, F-box genes; blue color, S-locus linked F-box genes 17

Supplementary Figure 14. Hybridization hotspots along each chromosome of the wolfberry genome 18

Supplementary Figure 15. The density distribution of the number of heterozygosity sites in each 200-kb sliding windows. The vertical line shows cutoff line with 1000 heterozygous sites (0.5% heterozygosity rate) 19

Supplementary Figure 16. Distribution of heterozygosity sites in each chromosome. 20

Supplementary Figure 17. The number of heterozygosity sites per hotspot along each chromosome, showing the highest level of heterozygosity in chromosome 2 (arrow). 21

Supplementary Figure 18. GO enrichments for the genes in hybridization hotspot along wolfberry genome. 22

Supplementary Figure 19. Phylogenetic analysis of type II MADS-box genes from *L. barbarum*, *S. lycopersicon*, *S. tuberosum*, *A. tricopoda* and *A. thaliana*.. 23

Supplementary Figure 20. The genes of expression profiles in floral and fruit development of *L. barbarum*. 24

Supplementary Figure 21. Comparison of transcriptional regulators of fleshy fruit development and ripening between tomato and *L. barbarum*. 25

Supplementary Figure 22. Phylogenetic analysis of the *Lycium LC* gene and its homologous genes.. 26

Supplementary Figure 23. Co-linear alignment of *Solanum lycopersicum* and *L. barbarum*. 27

Supplementary Figure 24. Phylogenetic analysis of the *Lycium CNR* gene and its homologous genes. 28

Supplementary Figure 25. Co-linear alignment of *Solanum lycopersicum* and *L. barbarum.* The red color of genes in the alignment denotes the tomato *CNR* and putative *Lycium CNR*. 29

Supplementary Figure 26. Possible candidate genes for *L. barbarum* polysaccharide (LBP) biosynthesis in *Lycium* fruits. 30

Supplementary Figure 27. The anthocyanin biosynthesis pathway. 32

Supplementary Figure 28. The carotenoid accumulation model in *L. barbarum*. 33

Supplementary Figure 29. Gene structure prediction results statistics. *L. barbarum* compared with genetic elements of related species. 34

Supplementary Tables 35

Supplementary Table 1. Summary of sequencing of *Lycium* species. 35

Supplementary Table 2. Assembly statistics of the *L. barbarum* haploid genome and *L. ruthenicum* diploid genome. 36

Supplementary Table 3. Genome assembly assessment of *L. barbarum* and *L. ruthenium* by BUSCO. 37

Supplementary Table 4. The chromosome length of *L. barbarum* by Hi-C. 38

Supplementary Table 5. Assembly statistics of the *L. barbarum* genome by Hi-C. 39

Supplementary Table 6. Prediction of gene structures of the *L. barbarum* and *L. ruthenicum* genome. 40

Supplementary Table 7. BUSCO assessment of *L. barbarum* and *L. ruthenicum* genome annotation. 41

Supplementary Table 8. Non-coding RNA annotation results of *L. barbarum* and *L. ruthenicum* genome. 42

Supplementary Table 9. The genes involved in anthocyanin biosynthesis pathway. 43

Supplementary Table 10. Gene expression levels (FPKMs) of anthocyanin biosynthesis-related in 5 stages along the *Lycium* fruit ripening. 44

Supplementary Table 11. Gene expression levels (FPKMs) of carotenoid biosynthesis-related in 5 stages along the *Lycium* fruit ripening. 45

Supplementary Table 12. Dating of whole genome triplication event. 46

Supplementary References 47

Supplementary Notes

Supplementary Note 1. Preparation of haploid sequencing samples

The haploid genome of *Lycium barbarum* was obtained by in vitro pollen culture. Diploid *L. barbarum* immature flower buds were used as test material and sterilized using 75% alcohol and 0.1% $HgCl_2$. This development stage ensured that the microspores were at the uninucleate or the very early binucleate stage. The anthers were stripped from the buds and inoculated in Murashige and Skoog (MS) medium (5% sucrose, 0.7% agar, 0.8% activated carbon, 1.0 mg/L 6-benzyladenine (6-BA) and 0.1 mg/L α-naphthaleneacetic acid (NAA) at 26°C ± 1°C, a for 12 h (L:D) photoperiod and light intensity of 2000 Lux. When the embryoid body grew small leaves, the seedlings were transferred to the medium (half-strength MS, 0.01 mg/L 6-BA and 0.2 mg/L NAA) for producing roots. The ploidy of the plants was detected by flow cytometry, and the haploid chromosome number was identified by root tip cells. Compared to a diploid plant, the haploid plant showed weak plant with smaller leaves and flowers; the anther lacked pollen (**Extended Data Figure 1**).

Supplementary Note 2. Gene family identification and phylogenomic dating

We downloaded genome and annotation data of *Antirrhinum majus* (http://bioinfo.sibs.ac.cn/Am ， version 1.0), *Arabidopsis thaliana* (TAIR 10), *Capsicum annuum* (GenBank accession number GCF_000710875.1), *Coffea arabica* (GenBank accession number GCF_003713225.1), *Cuscuta australis* (http://groups.english.kib.cas.cn/epb/dgd/Download/201711/t20171101_386248.html), *Helianthus annuus* (GenBank accession number GCF_002127325.1), *Ipomoea nil* (GenBank accession number GCF_001879475.1), *Nicotiana tabacum* (GenBank accession number GCF_000715135.1)， *Oryza sativa* (Ensembl, release-42), *Petunia axillaris* (https://solgenomics.net/organism, *Petunia axillaris* v1.6.2), *Petunia inflata* (https://solgenomics.net/organism, *Petunia inflata* v1.0.1), *Phalaenopsis equestris* (GenBank accession number GCF_001263595.1), *Sesamum indicum* (GenBank accession number GCF_000512975.1, *Solanum lycopersicum* (phytozome, ITAG2.4), *Solanum melongena* (http://eggplant.kazusa.or.jp), *Solanum tuberosum* (phytozome, v4.03) and *Vitis vinifera* (phytozome, Genoscope.12X). We chose the longest transcript to represent each gene. Gene family clustering was performed using OrthoMCL[1] based on the set of 38,984 predicted genes of *L. ruthenicum*, 33,581 predicted genes of *L. barbarum*, and the protein sets of the above 17 remaining plant species. This analysis yielded 17,478 gene families in *L. barbarum* containing 25,293 predicted genes (75.3% of the total genes identified), 17,331 gene families in *L. ruthenicum* containing 33,829 genes (86.8% of the total genes identified). (http://groups.english.kib.cas.cn/epb/dgd/Download/201711/t20171101_386248.html)

There are 259 single-copy gene families in all of the above 19 species. Therefore, we constructed a phylogenetic tree based on a concatenated sequence alignment of 259 extended

single-copy gene families using Phyml[2] software with the HKY85 model. Divergence times were estimated by PAML MCMCTREE[3]. The Markov chain Monte Carlo (MCMC) process was run for 500000 iterations with a sample frequency of 50 after a burn-in of 50000 iterations. Other parameters used the default settings of MCMCTREE. Two independent runs were performed to check convergence. The following constraints were used for time calibrations, which were all searched for in Timetree[4]: (i) the *Oryza sativa* and *C. canephor* divergence time (148–173 Mya); (ii) the *Vitis vinifera* and *C. canephor* divergence time (110–124 Mya); (iii) the *Sesamum indicum* and *C. canephor* divergence time (77–91 Mya); and (iv) the *S. tuberosum* and *S. lycopersicum* divergence time (5–9 Mya).

Supplementary Figures

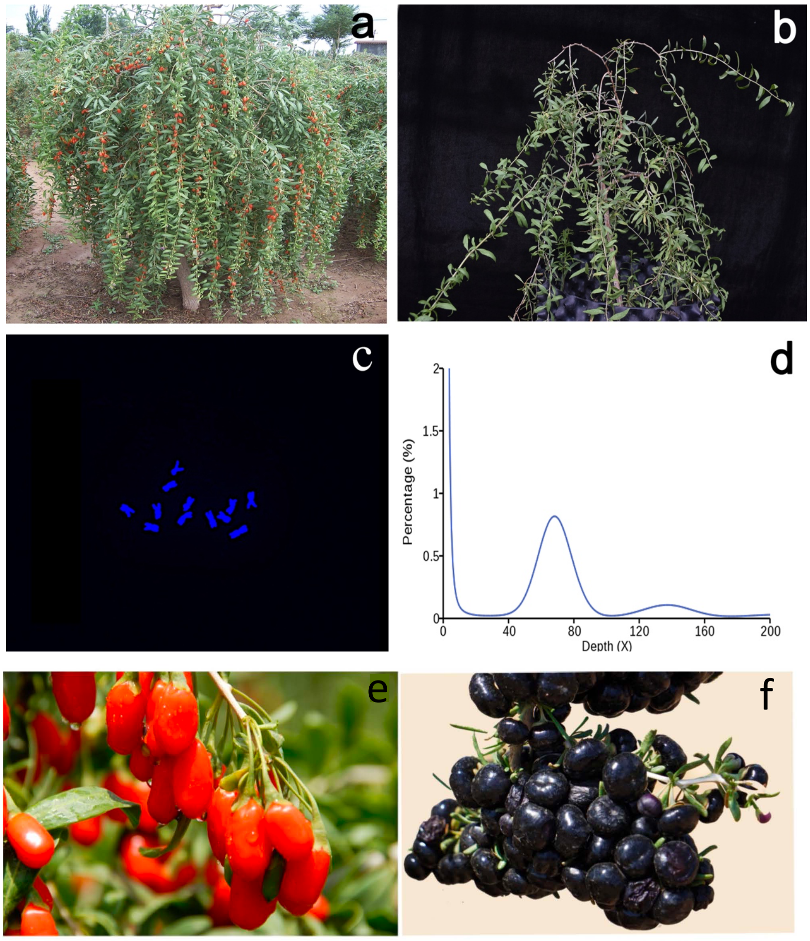

Supplementary Figure 1. Cytogenetic analysis and comparison of haploid and diploid plants of *L. barbarum*. a. Diploid plant. **b.** Haploid plant. **c.** Haploid plant with 12 chromosomes. **d.** *K-mer* analysis of a haploid plant with one peak and little heterozygosity. **e.** Red fruit of *L. barbarum*. **f.** Black fruit of *L. ruthenicum*.

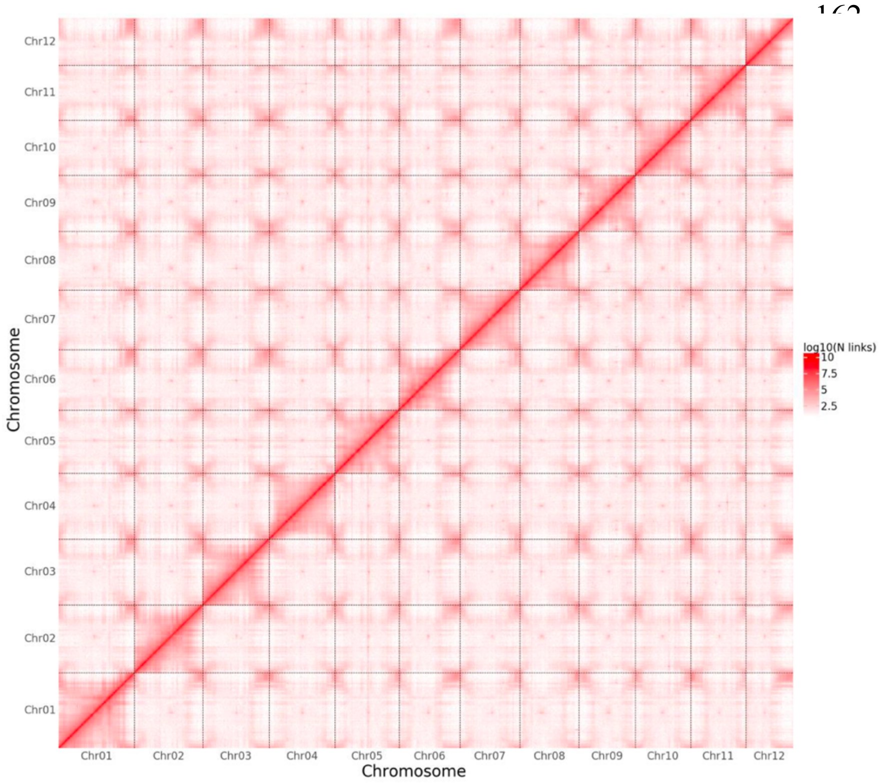

Supplementary Figure 2. Intensity signal heat map of Hi-C chromosome interaction of *L. barbarum* genome.

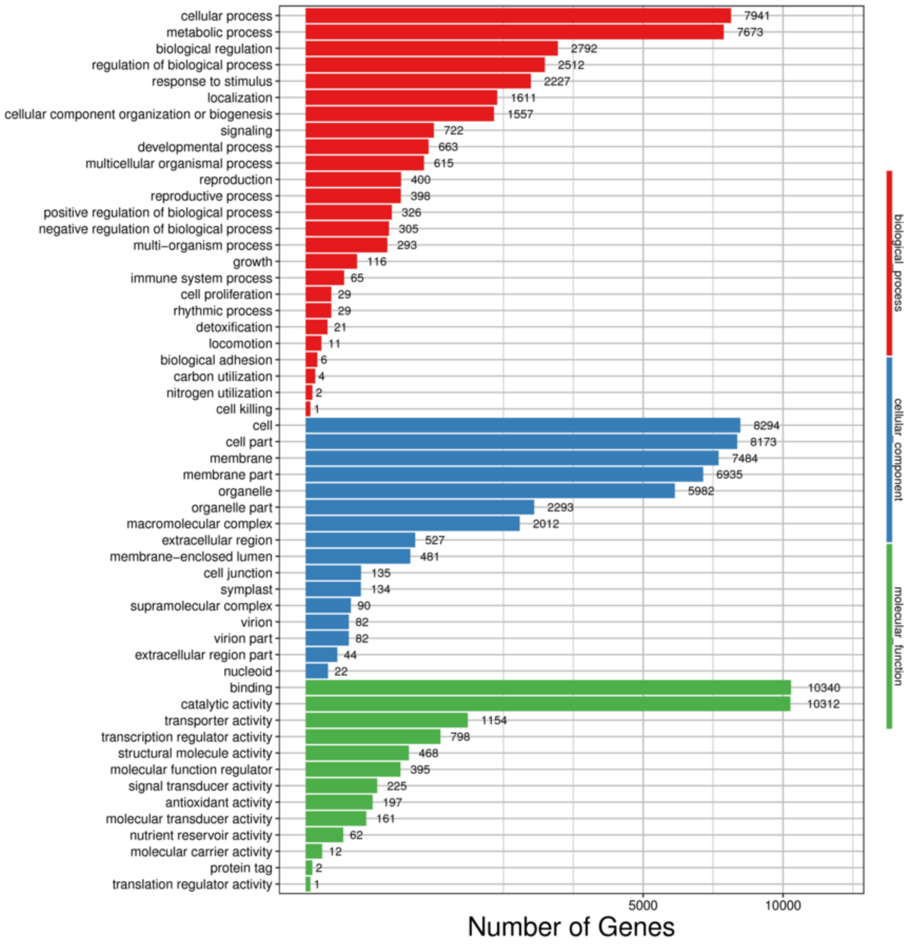

Supplementary Figure 3. GO secondary functional enrichment for the expanded gene families in *L. barbarum*.

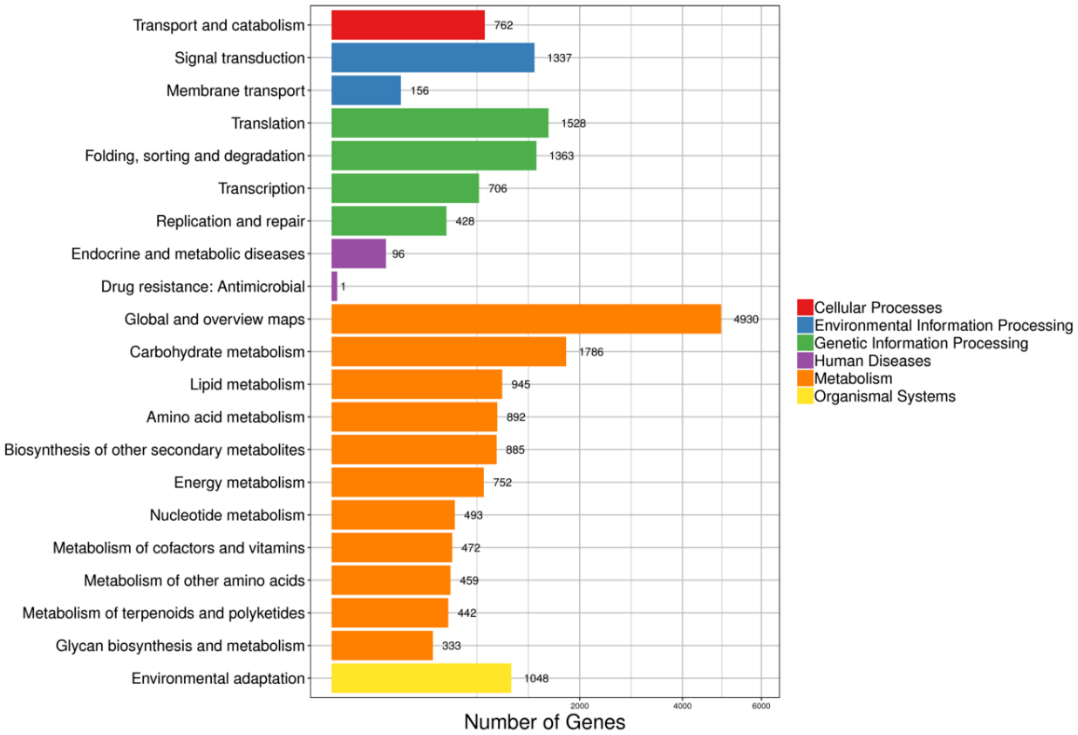

Supplementary Figure 4. KEGG pathway functional enrichment for the expanded gene families in *L. barbarum*.

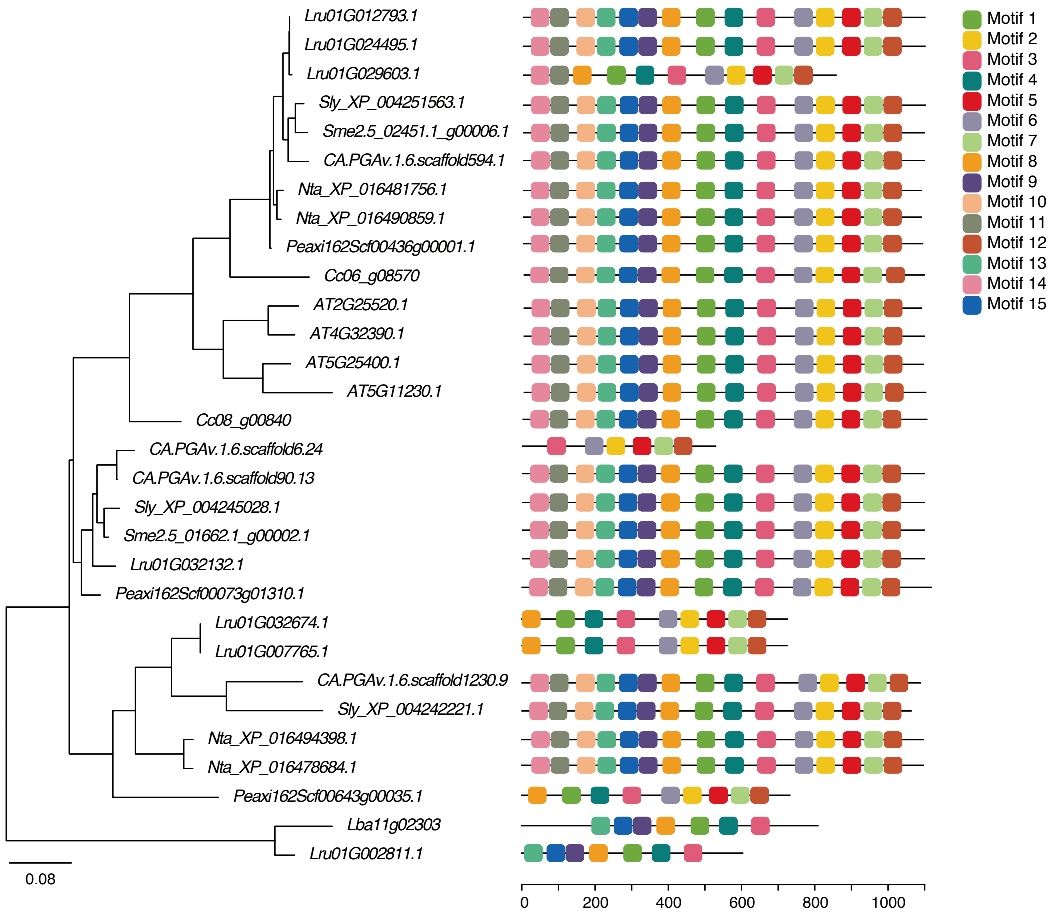

Supplementary Figure 5. Phylogenetic tree of genes involved in triose phosphate/phosphate translocator (TPT) in Solanaceae. The motif M10-M13-M15-M9 presents a variation in *Lycium* species.

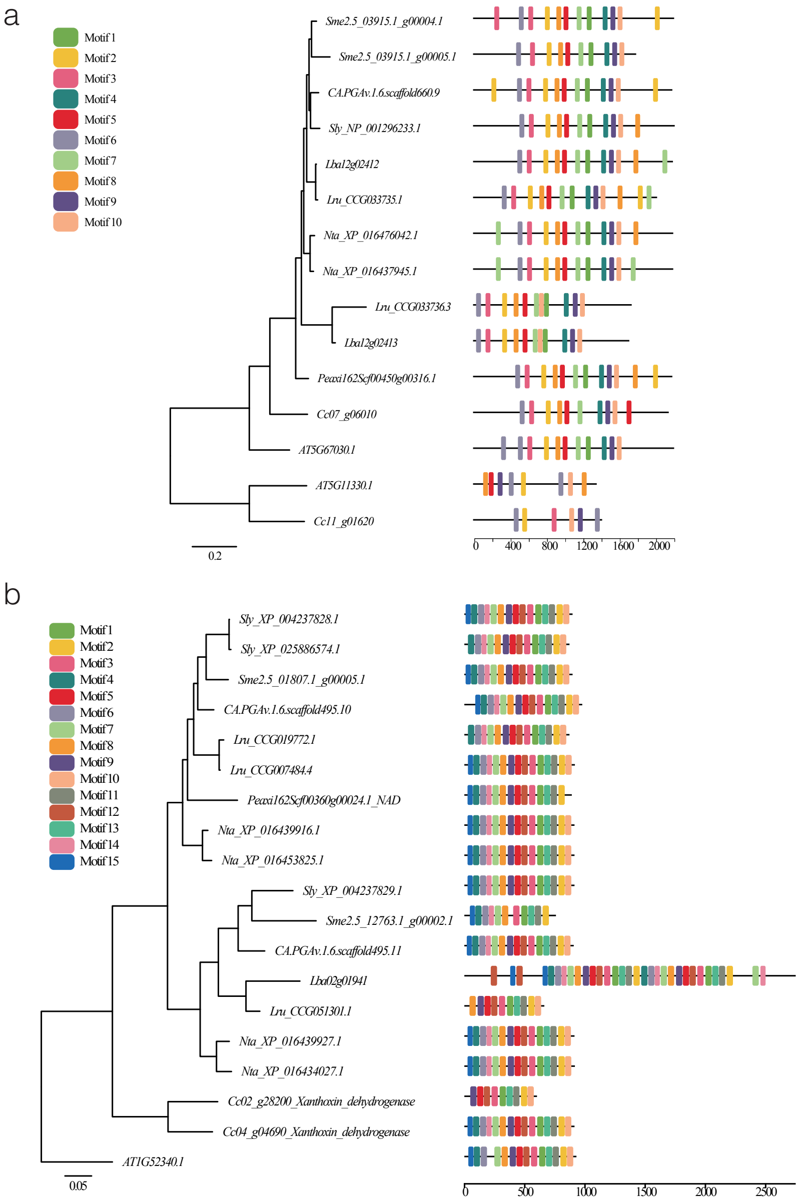

Supplementary Figure 6. Phylogenetic tree of genes involved in zeaxanthin epoxidase and xanthoxin oxidase in Solanaceae. a. Phylogenetic tree of genes involved in zeaxanthin epoxidase in Solanaceae. *Lycium* shows a genus-specific expansion and resulted in an M10 insertion between M1 and M7. **b.** Phylogenetic tree of genes involved in xanthoxin oxidase in Solanaceae. Solanaceae shows a family-specific duplication, while the homologous genes of *Lycium* in the second clade show a dramatic sequence rearrangement.

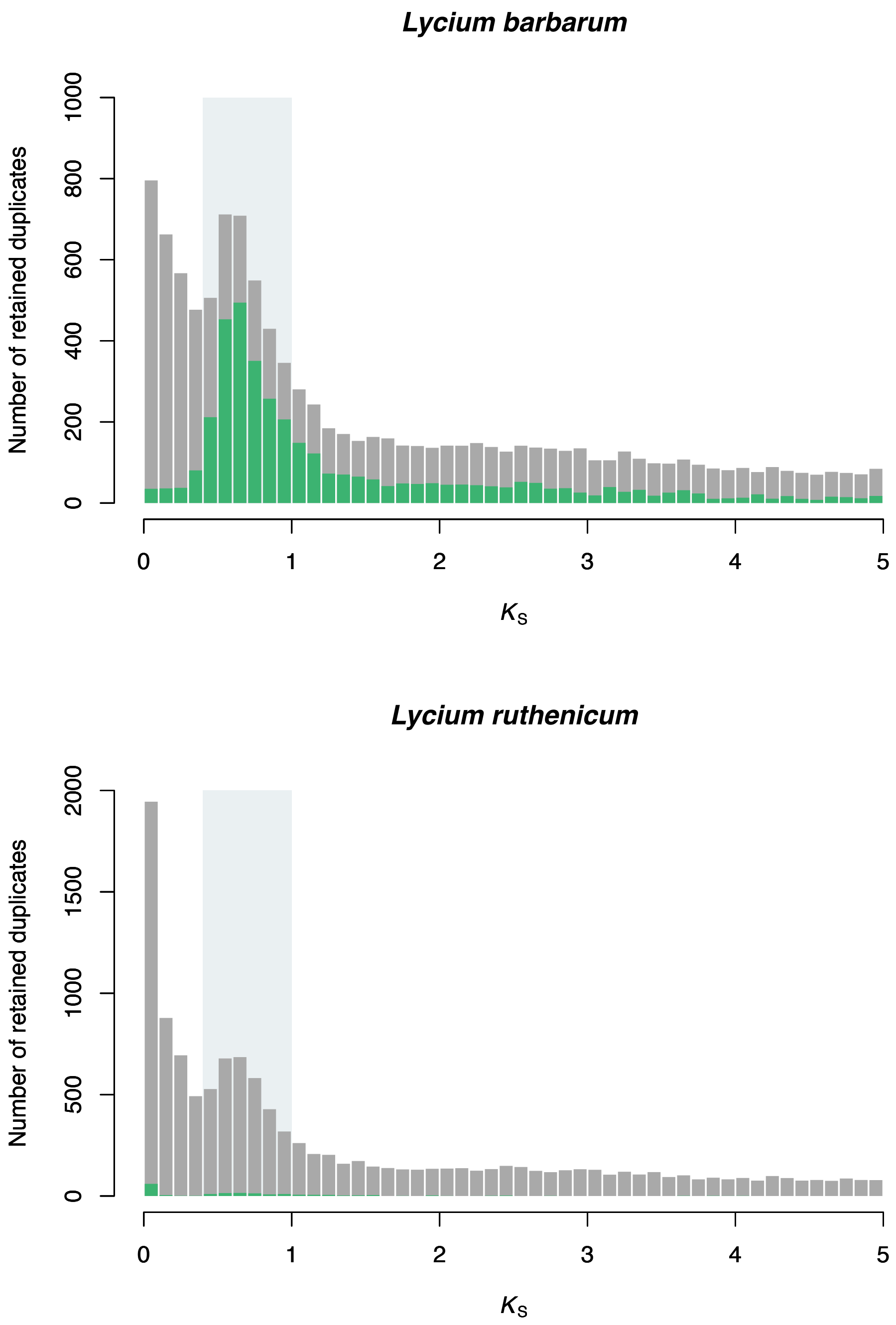

Supplementary Figure 7. K_S age distributions for the whole paranome (grey) and anchor pairs (green) in *L. barbarum* and *L. ruthenicum*. Both K_S distributions show a peak at $K_S \approx 0.65$ falling in a K_S range highlighted by the light grey rectangles in the from 0.4 to 1.

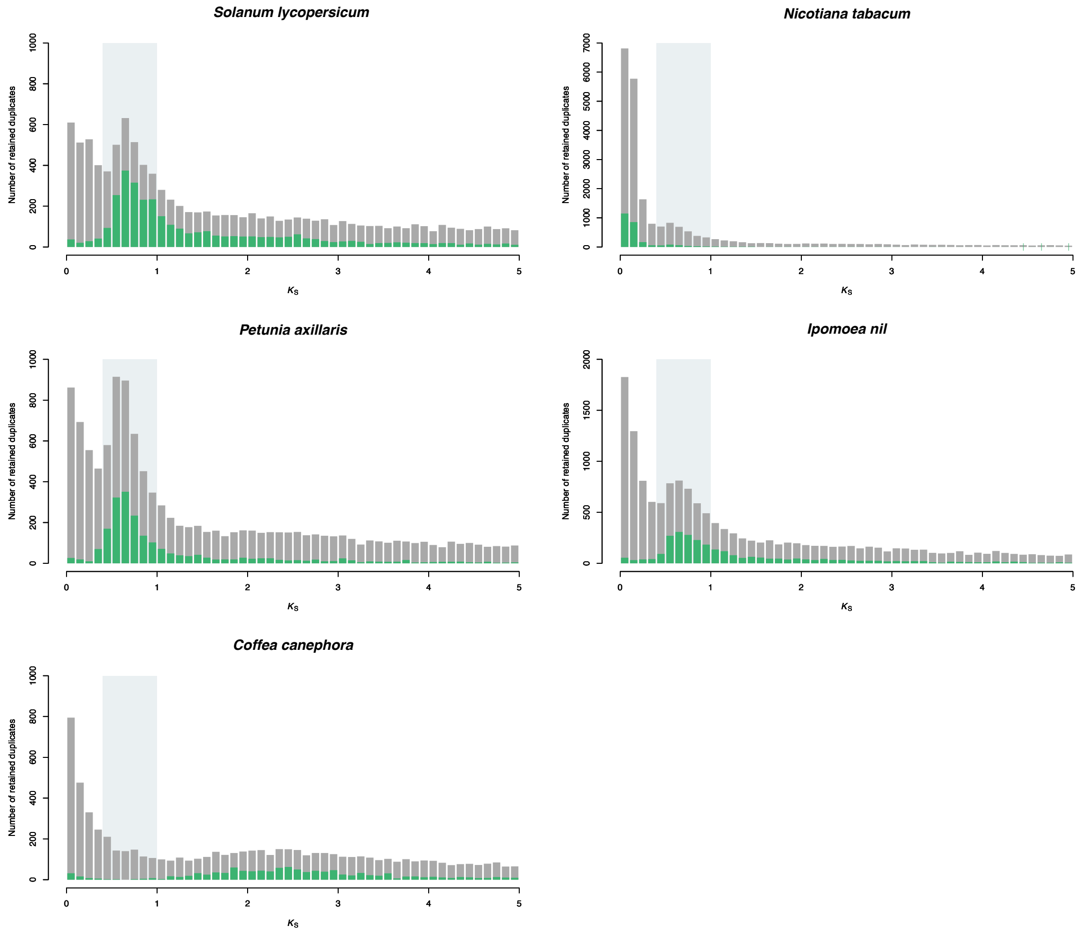

Supplementary Figure 8. K_S age distributions for the whole paranome (grey) and anchor pairs (green) in several other species from Solanaceae and Convolvulaceae and *C. canephora*. The light grey rectangles highlight a K_S range from 0.4 to 1 as identified in the two *Lycium* genomes **(Figure 2 and Supplementary Figure 7).** Please note that *N. tabacum* is an allopolyploid species.

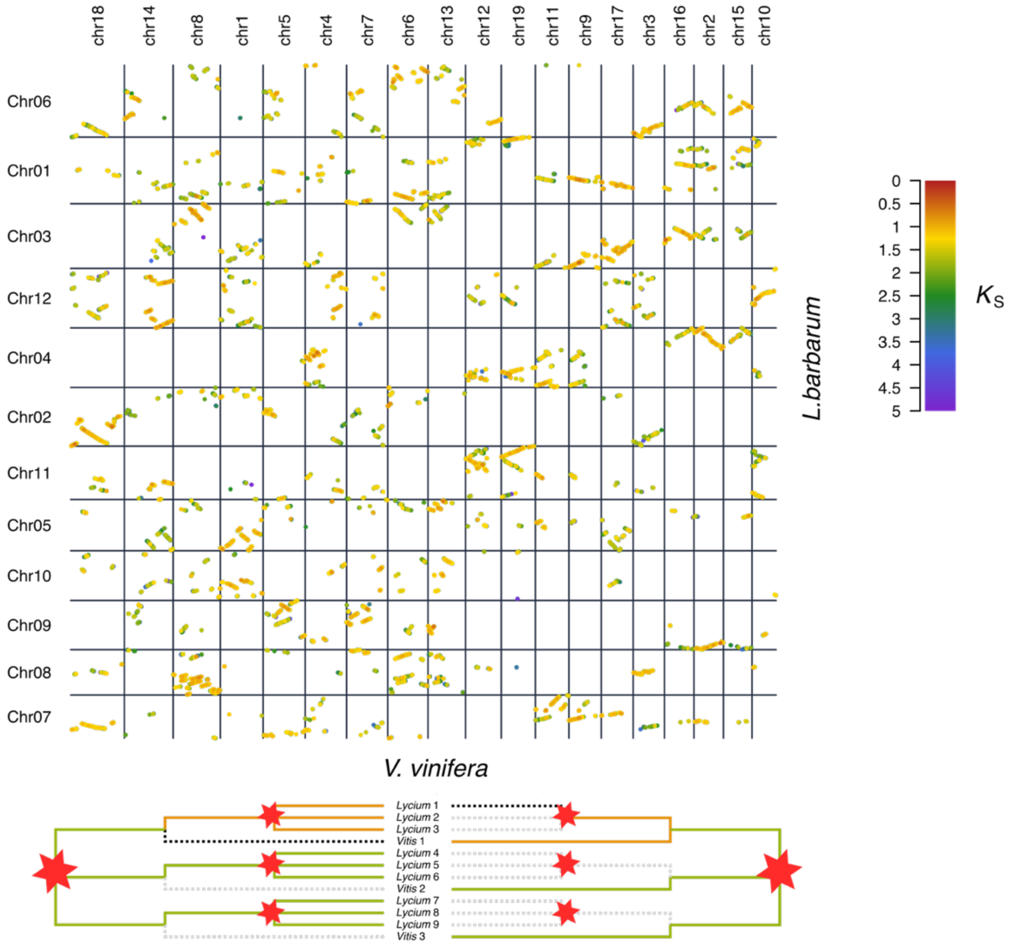

Supplementary Figure 9. Dot plot for inter-genomic comparison between *L. barbarum* and *V. vinifera*. Only collinear blocks with at least five anchor pairs are shown (see Methods). The colours of dots denote K_S values between anchor pairs. The phylogenetic trees below the dot plot are aids to interpret the dot plot with the expected outcomes in *Lycium* and *Vitis* after the hexaploidization shared by core eudicots and the hexaploidization in Solanaceae. Using a genomic segment in *V. vinifera* as a reference, such as *Vitis* 1 (black dotted line in the left-hand tree), it is expected that up to three collinear segments with smaller K_S values (orange) and up to six collinear segments with larger K_S values (green) can be observed in *L. barbarum* (x-axis and reading the dot plot vertically). Alternatively, using a genomic segment in *L. barbarum* as a reference, such as *Lycium* 1 (black dotted line in the right-hand tree), it is expected that up to one collinear segment with smaller K_S values (orange) and up to two collinear segments with larger K_S values (green) can be observed in *V. vinifera* (y-axis and reading the dot plot horizontally). Dotted branches on both trees denote the segments that should read from the x-axis (left-hand tree) or the y-axis (right-hand tree). The hexagrams denote the hexaploidization events.

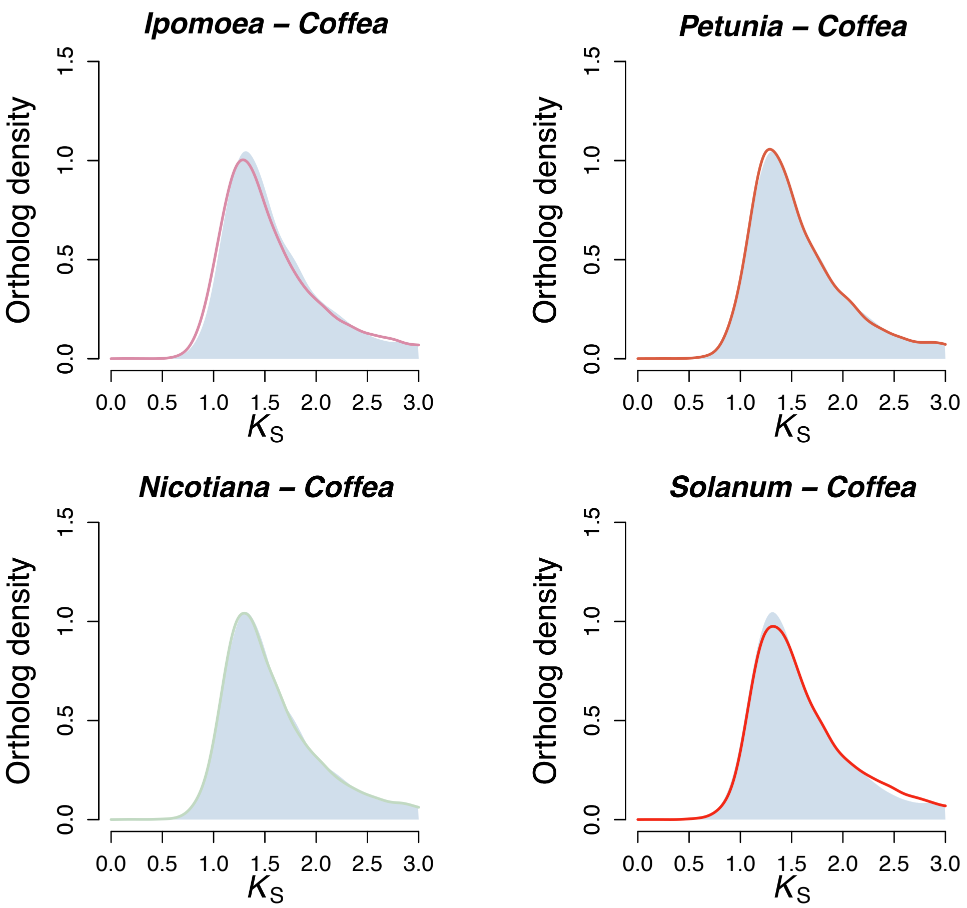

Supplementary Figure 10. Distributions of synonymous substitutions per synonymous site (*K_S*) of one-to-one orthologs identified between species from Solanaceae and Convolvulaceae and *C. canephora*. In each plot, the kernel density estimation (KDE) of an orthologous K_S distribution between *L. barbarum* and *C. canephora* (filled curve) is compared with the KDE curves of the orthologous K_S distribution between *C. canephora* and *I. nil*, *P. axillaris*, *N. tabacum*, and *S. lycopersicum* (coloured lines), respectively.

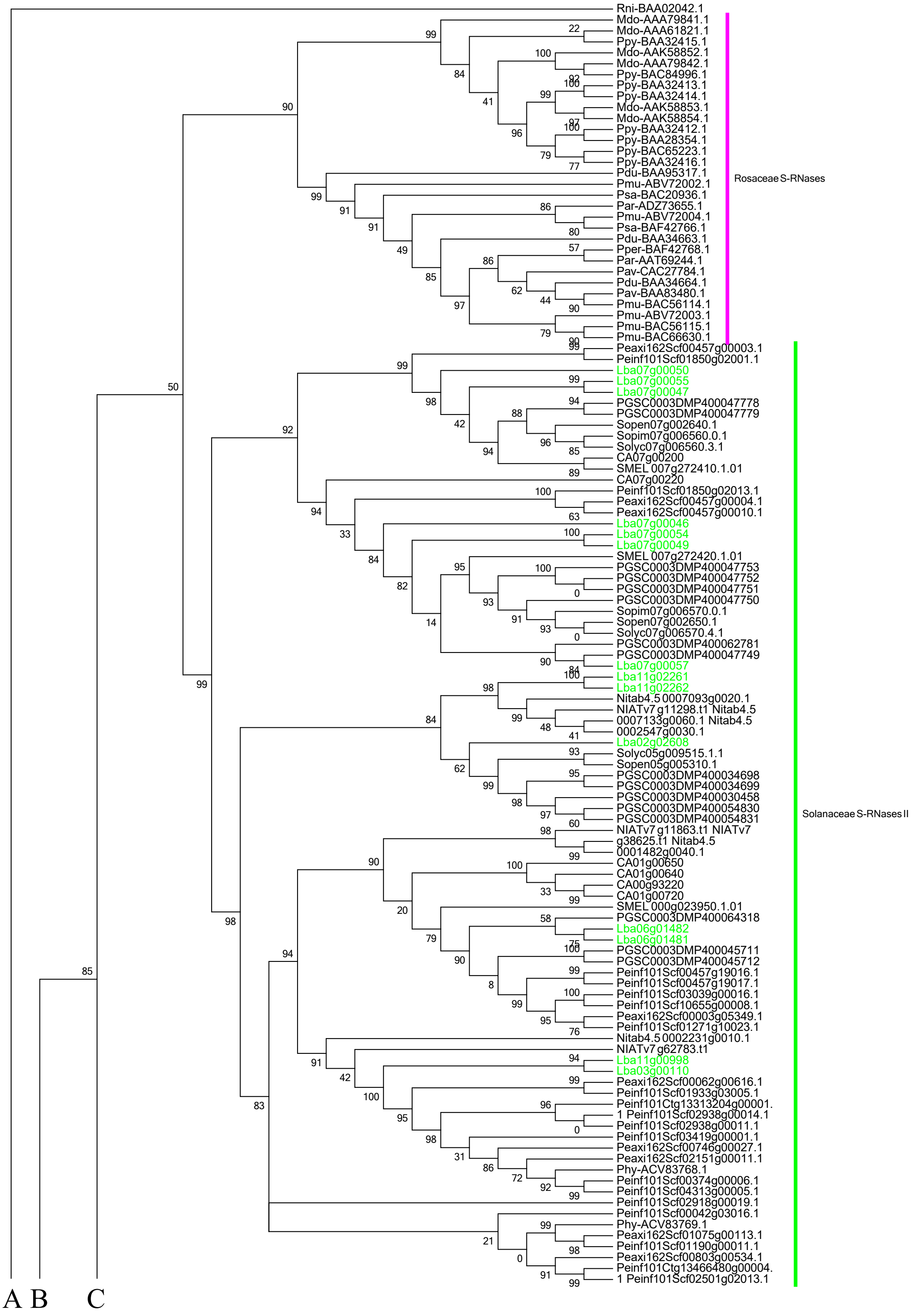
Rni-BAA02042.1
Mdo-AAA79841.1
Mdo-AAA61821.1
Ppy-BAA32415.1
Mdo-AAK58852.1
Mdo-AAA79842.1
Ppy-BAC84996.1
Ppy-BAA32413.1
Ppy-BAA32414.1
Mdo-AAK58853.1
Mdo-AAK58854.1
Ppy-BAA32412.1
Ppy-BAA28354.1
Ppy-BAC65223.1
Ppy-BAA32416.1
Pdu-BAA95317.1
Pmu-ABV72002.1
Psa-BAC20936.1
Par-ADZ73655.1
Pmu-ABV72004.1
Psa-BAF42766.1
Pdu-BAA34663.1
Pper-BAF42768.1
Par-AAT69244.1
Pav-CAC27784.1
Pdu-BAA34664.1
Pav-BAA83480.1
Pmu-BAC56114.1
Pmu-ABV72003.1
Pmu-BAC56115.1
Pmu-BAC66630.1
Peaxi162Scf00457g00003.1
Peinf101Scf01850g02001.1
Lba07g00050
Lba07g00055
Lba07g00047
PGSC0003DMP400047778
PGSC0003DMP400047779
Sopen07g002640.1
Sopim07g006560.0.1
Solyc07g006560.3.1
CA07g00200
SMEL 007g272410.1.01
CA07g00220
Peinf101Scf01850g02013.1
Peaxi162Scf00457g00004.1
Peaxi162Scf00457g00010.1
Lba07g00046
Lba07g00054
Lba07g00049
SMEL 007g272420.1.01
PGSC0003DMP400047753
PGSC0003DMP400047752
PGSC0003DMP400047751
PGSC0003DMP400047750
Sopim07g006570.0.1
Sopen07g002650.1
Solyc07g006570.4.1
PGSC0003DMP400062781
PGSC0003DMP400047749
Lba07g00057
Lba11g02261
Lba11g02262
Nitab4.5 0007093g0020.1
NIATv7 g11298.t1 Nitab4.5
0007133g0060.1 Nitab4.5
0002547g0030.1
Lba02g02608
Solyc05g009515.1.1
Sopen05g005310.1
PGSC0003DMP400034698
PGSC0003DMP400034699
PGSC0003DMP400030458
PGSC0003DMP400054830
PGSC0003DMP400054831
NIATv7 g11863.t1 NIATv7
g38625.t1 Nitab4.5
0001482g0040.1
CA01g00650
CA01g00640
CA00g93220
CA01g00720
SMEL 000g023950.1.01
PGSC0003DMP400064318
Lba06g01482
Lba06g01481
PGSC0003DMP400045711
PGSC0003DMP400045712
Peinf101Scf00457g19016.1
Peinf101Scf00457g19017.1
Peinf101Scf03039g00016.1
Peinf101Scf10655g00008.1
Peaxi162Scf00003g05349.1
Peinf101Scf01271g10023.1
Nitab4.5 0002231g0010.1
NIATv7 g62783.t1
Lba11g00998
Lba03g00110
Peaxi162Scf00062g00616.1
Peinf101Scf01933g03005.1
Peinf101Ctg13313204g00001.
1 Peinf101Scf02938g00014.1
Peinf101Scf02938g00011.1
Peinf101Scf03419g00001.1
Peaxi162Scf00746g00027.1
Peaxi162Scf02151g00011.1
Phy-ACV83768.1
Peinf101Scf00374g00006.1
Peinf101Scf04313g00005.1
Peinf101Scf02918g00019.1
Peinf101Scf00042g03016.1
Phy-ACV83769.1
Peaxi162Scf01075g00113.1
Peinf101Scf01190g00011.1
Peaxi162Scf00803g00534.1
Peinf101Ctg13466480g00004.
1 Peinf101Scf02501g02013.1
Rosaceae S-RNases
Solanaceae S-RNases II
A
B
C

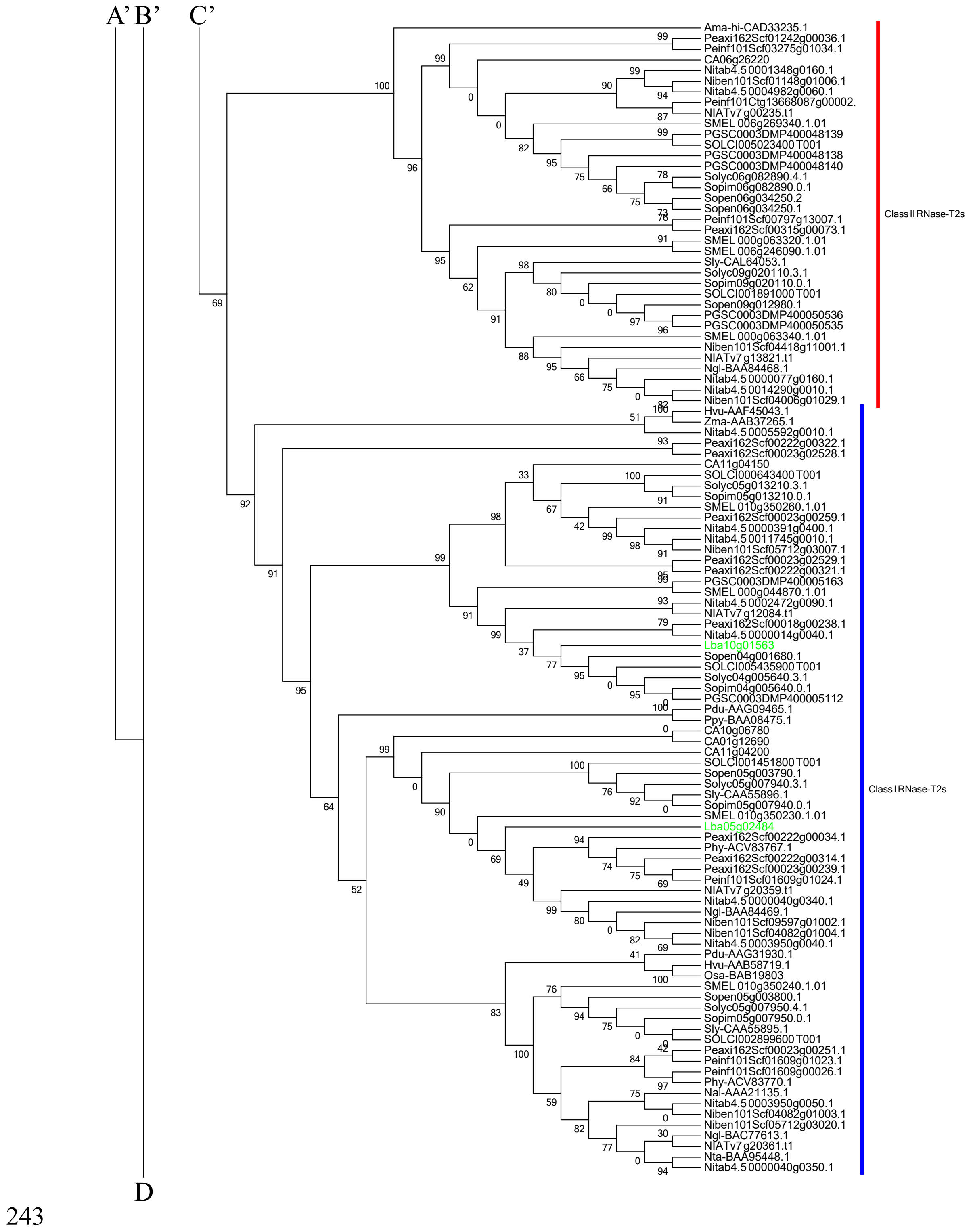
A’ B’ C’
Ama-hi-CAD33235.1
Peaxi162Scf01242g00036.1
Peinf101Scf03275g01034.1
CA06g26220
Nitab4.5 0001348g0160.1
Niben101Scf01148g01006.1
Nitab4.5 0004982g0060.1
Peinf101Ctg13668087g00002.
NIATv7 g00235.t1
SMEL 006g269340.1.01
PGSC0003DMP400048139
SOLCI005023400 T001
PGSC0003DMP400048138
PGSC0003DMP400048140
Solyc06g082890.4.1
Sopim06g082890.0.1
Sopen06g034250.2
Sopen06g034250.1
Peinf101Scf00797g13007.1
Peaxi162Scf00315g00073.1
SMEL 000g063320.1.01
SMEL 006g246090.1.01
Sly-CAL64053.1
Solyc09g020110.3.1
Sopim09g020110.0.1
SOLCI001891000 T001
Sopen09g012980.1
PGSC0003DMP400050536
PGSC0003DMP400050535
SMEL 000g063340.1.01
Niben101Scf04418g11001.1
NIATv7 g13821.t1
Ngl-BAA84468.1
Nitab4.5 0000077g0160.1
Nitab4.5 0014290g0010.1
Niben101Scf04006g01029.1
Class II RNase-T2s
Hvu-AAF45043.1
Zma-AAB37265.1
Nitab4.5 0005592g0010.1
Peaxi162Scf00222g00322.1
Peaxi162Scf00023g02528.1
CA11g04150
SOLCI000643400 T001
Solyc05g013210.3.1
Sopim05g013210.0.1
SMEL 010g350260.1.01
Peaxi162Scf00023g00259.1
Nitab4.5 0000391g0400.1
Nitab4.5 0011745g0010.1
Niben101Scf05712g03007.1
Peaxi162Scf00023g02529.1
Peaxi162Scf00222g00321.1
PGSC0003DMP400005163
SMEL 000g044870.1.01
Nitab4.5 0002472g0090.1
NIATv7 g12084.t1
Peaxi162Scf00018g00238.1
Nitab4.5 0000014g0040.1
Lba10g01563
Sopen04g001680.1
SOLCI005435900 T001
Solyc04g005640.3.1
Sopim04g005640.0.1
PGSC0003DMP400005112
Pdu-AAG09465.1
Ppy-BAA08475.1
CA10g06780
CA01g12690
CA11g04200
SOLCI001451800 T001
Sopen05g003790.1
Solyc05g007940.3.1
Sly-CAA55896.1
Sopim05g007940.0.1
SMEL 010g350230.1.01
Lba05g02484
Peaxi162Scf00222g00034.1
Phy-ACV83767.1
Peaxi162Scf00222g00314.1
Peaxi162Scf00023g00239.1
Peinf101Scf01609g01024.1
NIATv7 g20359.t1
Nitab4.5 0000040g0340.1
Ngl-BAA84469.1
Niben101Scf09597g01002.1
Niben101Scf04082g01004.1
Nitab4.5 0003950g0040.1
Pdu-AAG31930.1
Hvu-AAB58719.1
Osa-BAB19803
SMEL 010g350240.1.01
Sopen05g003800.1
Solyc05g007950.4.1
Sopim05g007950.0.1
Sly-CAA55895.1
SOLCI002899600 T001
Peaxi162Scf00023g00251.1
Peinf101Scf01609g01023.1
Peinf101Scf01609g00026.1
Phy-ACV83770.1
Nal-AAA21135.1
Nitab4.5 0003950g0050.1
Niben101Scf04082g01003.1
Niben101Scf05712g03020.1
Ngl-BAC77613.1
NIATv7 g20361.t1
Nta-BAA95448.1
Nitab4.5 0000040g0350.1
Class I RNase-T2s
D

243

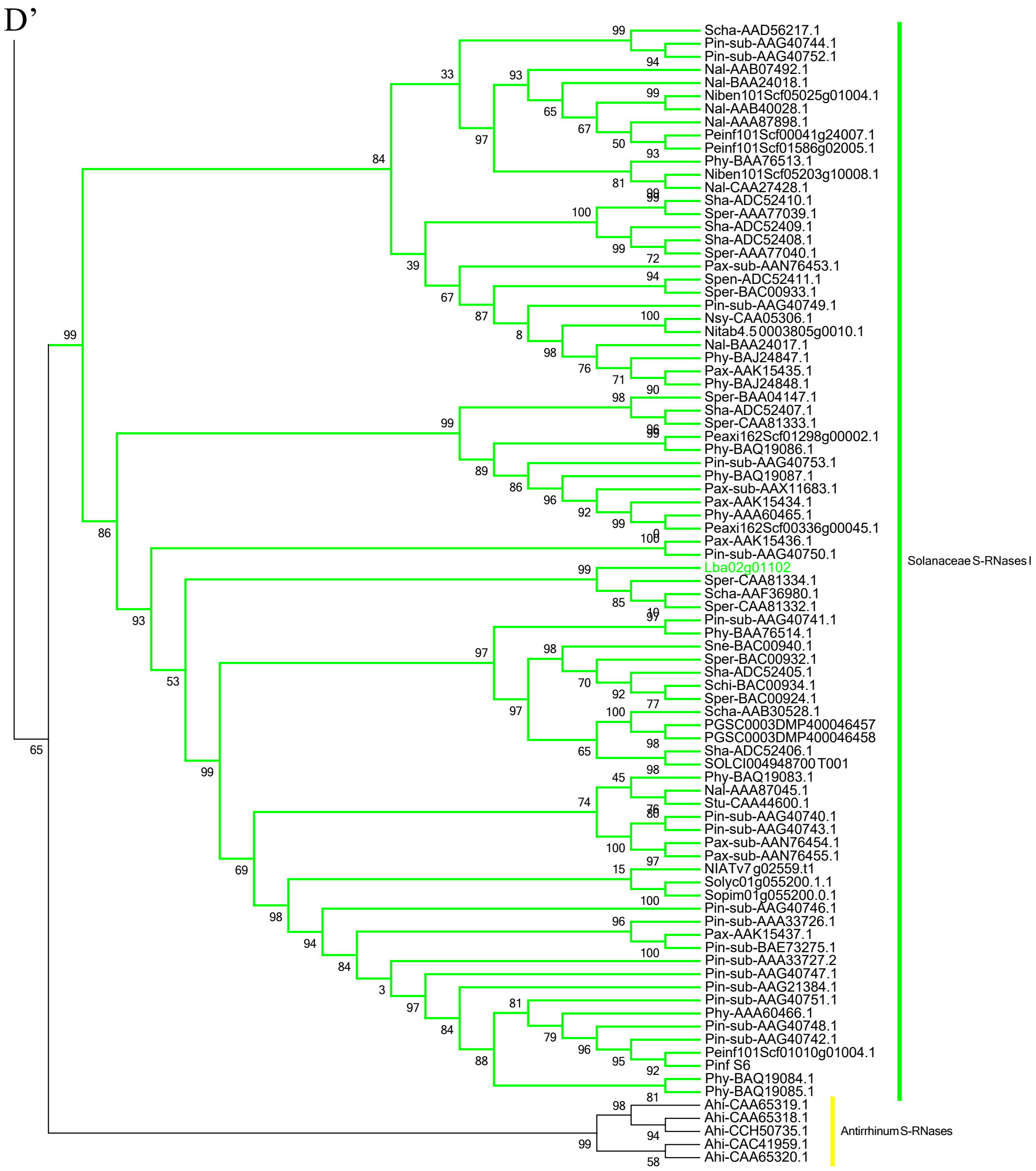

Supplementary Figure 11. Maximum likelihood phylogenetic tree of the RNase-T2-like genes obtained from wolfberry and S-RNase-related RNase-T2s obtained from species possessing S-RNase-based amino acids in Solanaceae.

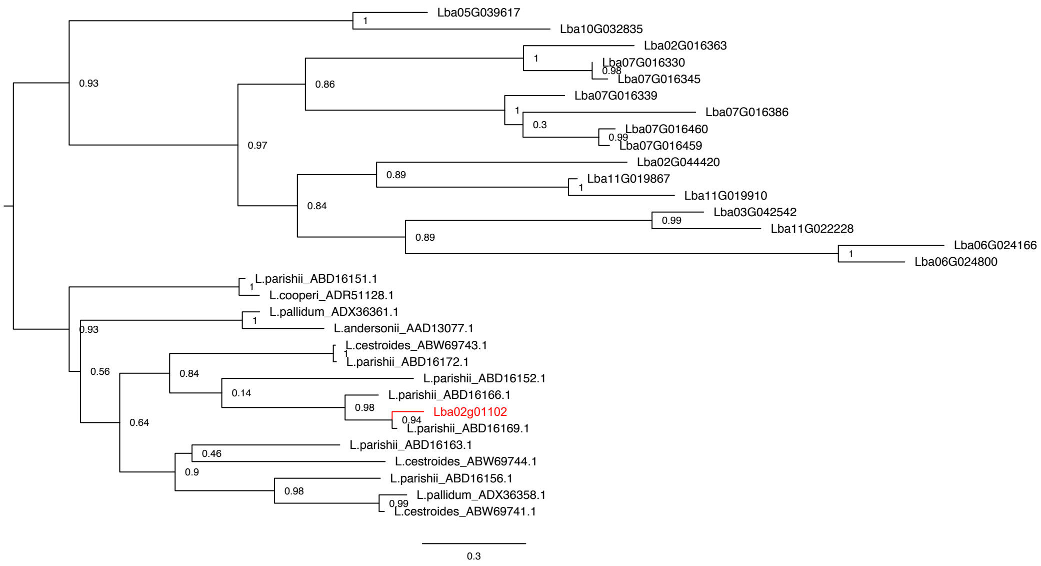

Supplementary Figure 12. Maximum likelihood phylogenetic tree of the RNase-T2 genes obtained from *L. barbarum* and S-RNase genes from *Lycium* species cited by Miller *et al*.[5].

The tree was constructed in PhyML 3.0. Red color showed SI candidate *Lba02g01102* gene.

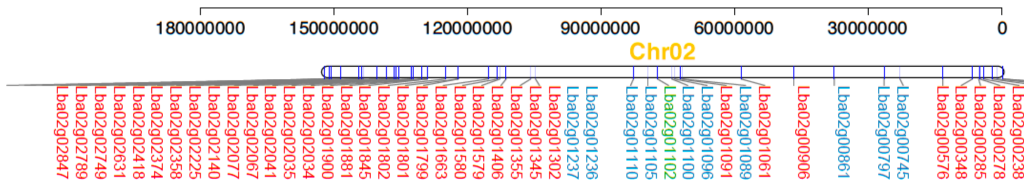

Supplementary Figure 13. YF-box genes distribution along chromosome 2 of *L. barbarum*.

Green color, SI candidate *Lba02g01102* gene; red color, F-box genes; blue color, S-locus linked F-box genes.

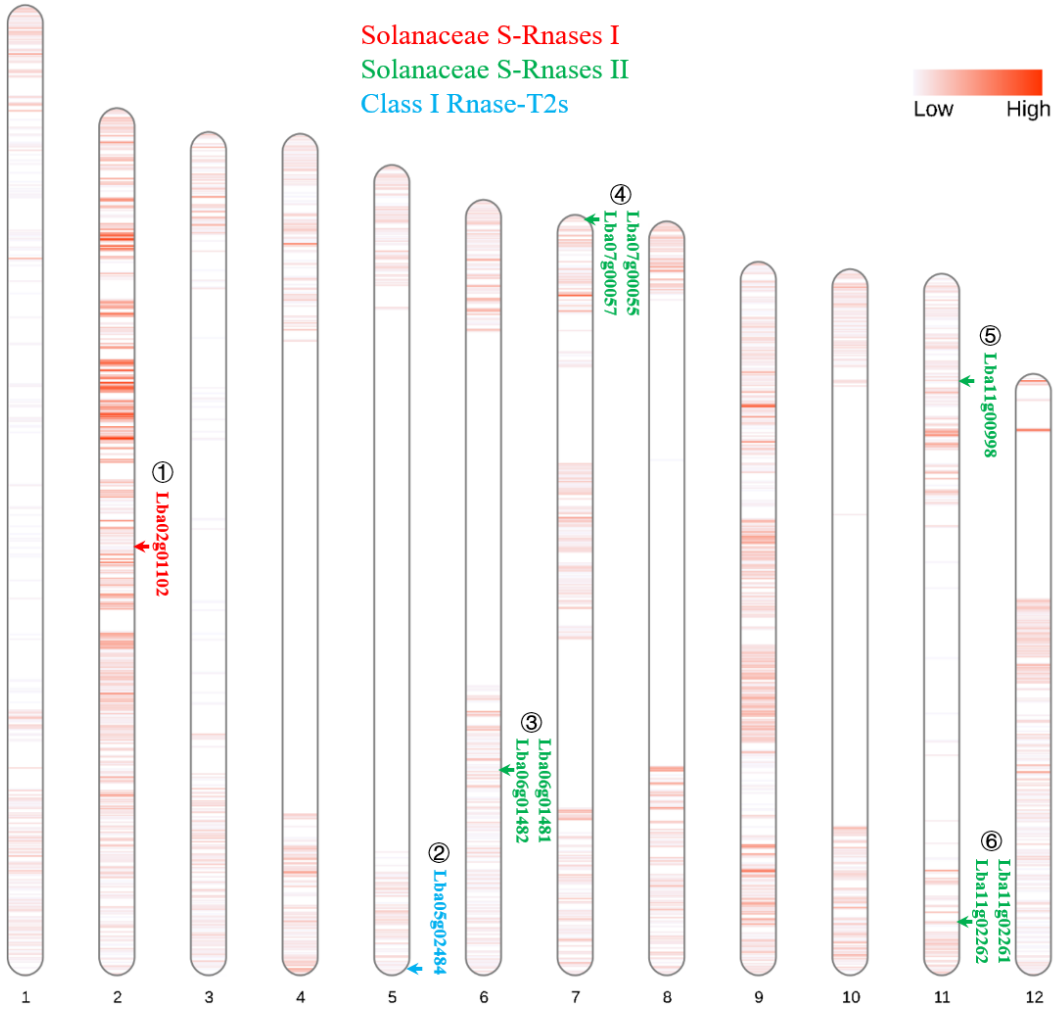

Supplementary Figure 14. Hybridization hotspots along each chromosome of the wolfberry genome. The blocks of hotspots were identified if the 200-kb sliding window has more than 1000 heterozygous sites. The RNases-T2 genes are labelled in each chromosome according to their groups (Solanaceae S-Rnases I, Solanaceae S-Rnases II, and Class I Rnase-T2s). Nine of them present in the six hybridization hotspots along chromosomes 2, 5, 6, 7, and 11 mainly function in RNase-T2 activity, with Solanaceae S-Rnases I genes belonging to hotspot ① (chr02: *Lba02g01102*); Solanaceae S-Rnases II genes belonging to hotspot ② (chr05: *Lba05g02484*); and Class I Rnase-T2s genes belonging to hotspots ③ (chr06: *Lba06g01481*, *Lba06g01482*); ④ (chr07: *Lba07g00055*, *Lba07g00057*); ⑤ (chr11: *Lba11g00998*); and ⑥ (chr11: *Lba11g02261*, *Lba11g02262*).

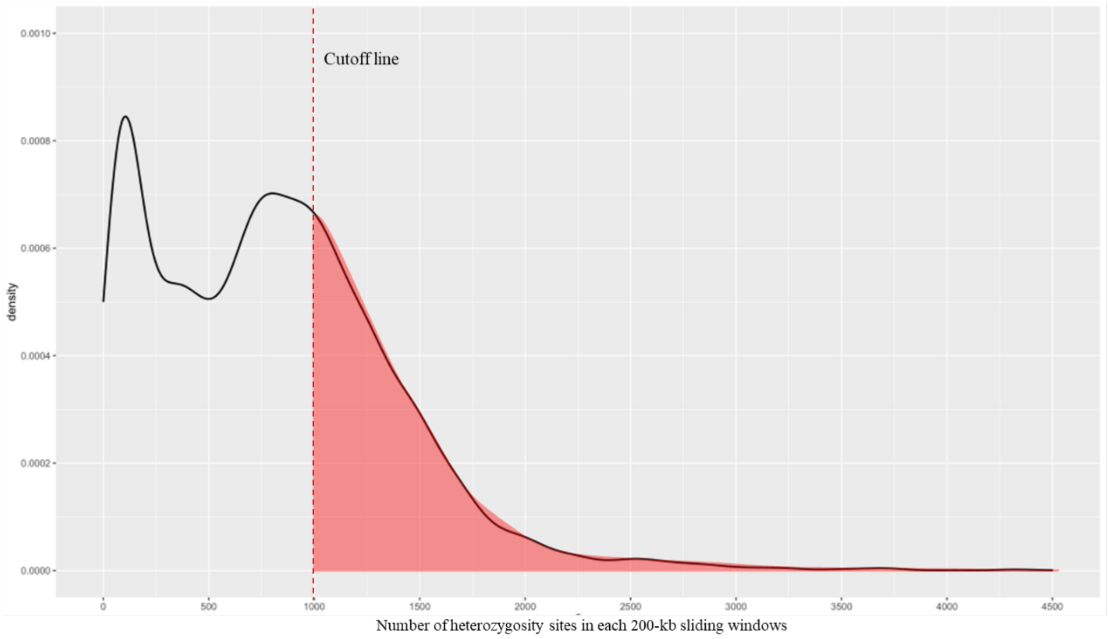

Supplementary Figure 15. The density distribution of the number of heterozygosity sites in each 200-kb sliding windows. The vertical line shows cutoff line with 1000 heterozygous sites (0.5% heterozygosity rate).

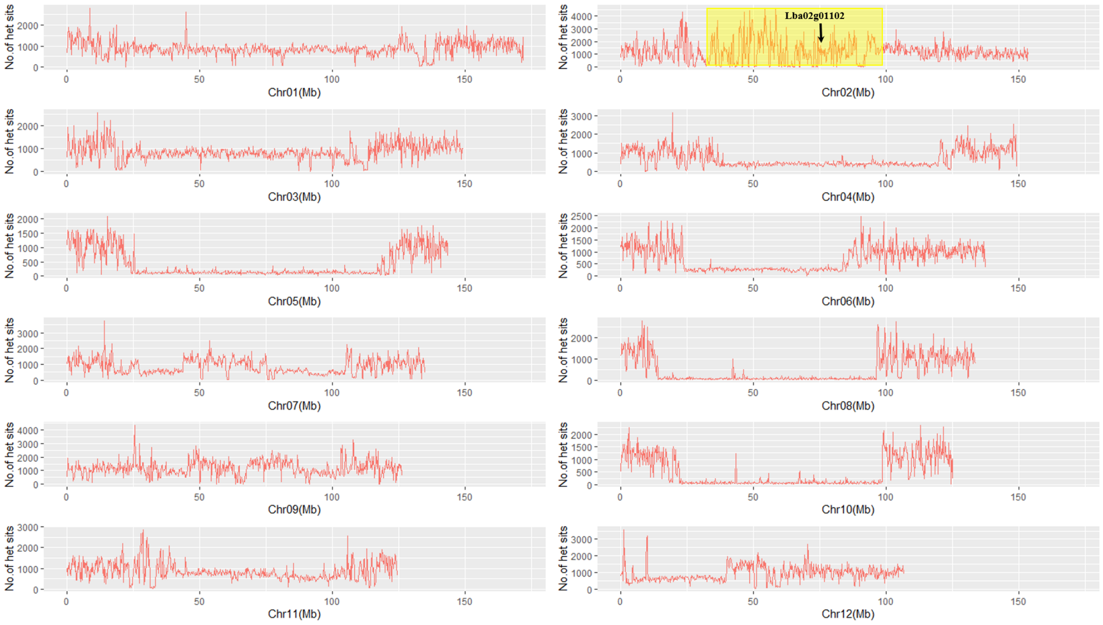

Supplementary Figure 16. Distribution of heterozygosity sites in each chromosome. The highlighted region shows elevated heterozygosity rates in the middle region of chromosome 2 around S-RNases I type SI candidate (*Lba02g01102*) compared with the corresponding regions of other chromosomes.

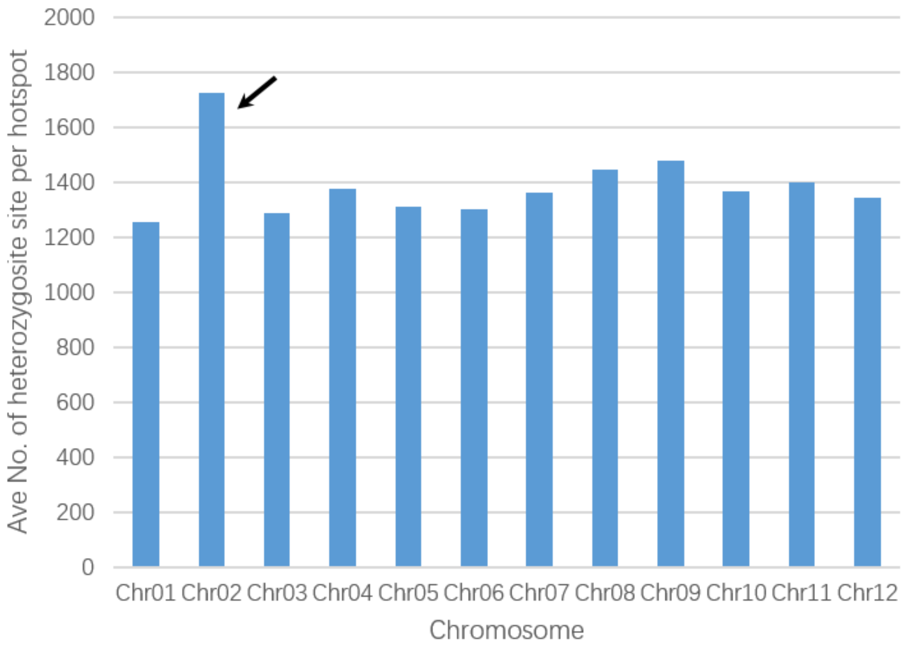

Supplementary Figure 17. The number of heterozygosity sites per hotspot along each chromosome, showing the highest level of heterozygosity in chromosome 2 (arrow).

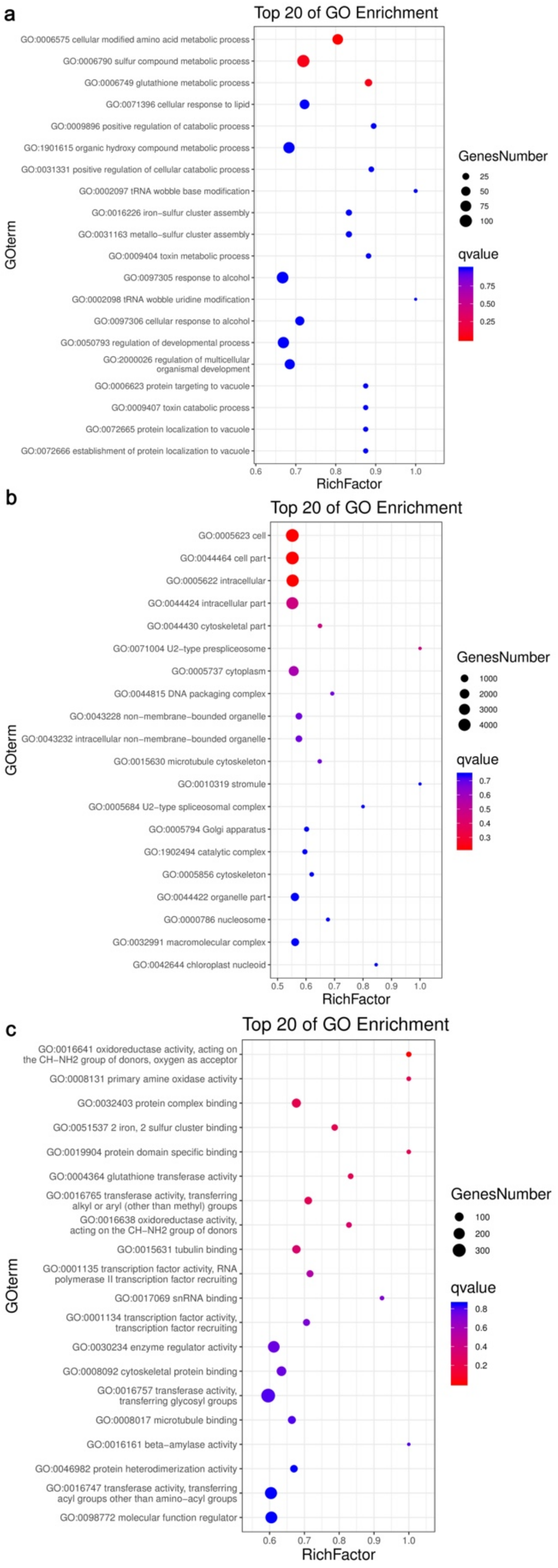

Supplementary Figure 18. GO enrichments for the genes in hybridization hotspot along wolfberry genome. a. Biological process; **b**. Cellular components; **c**. Molecular function.

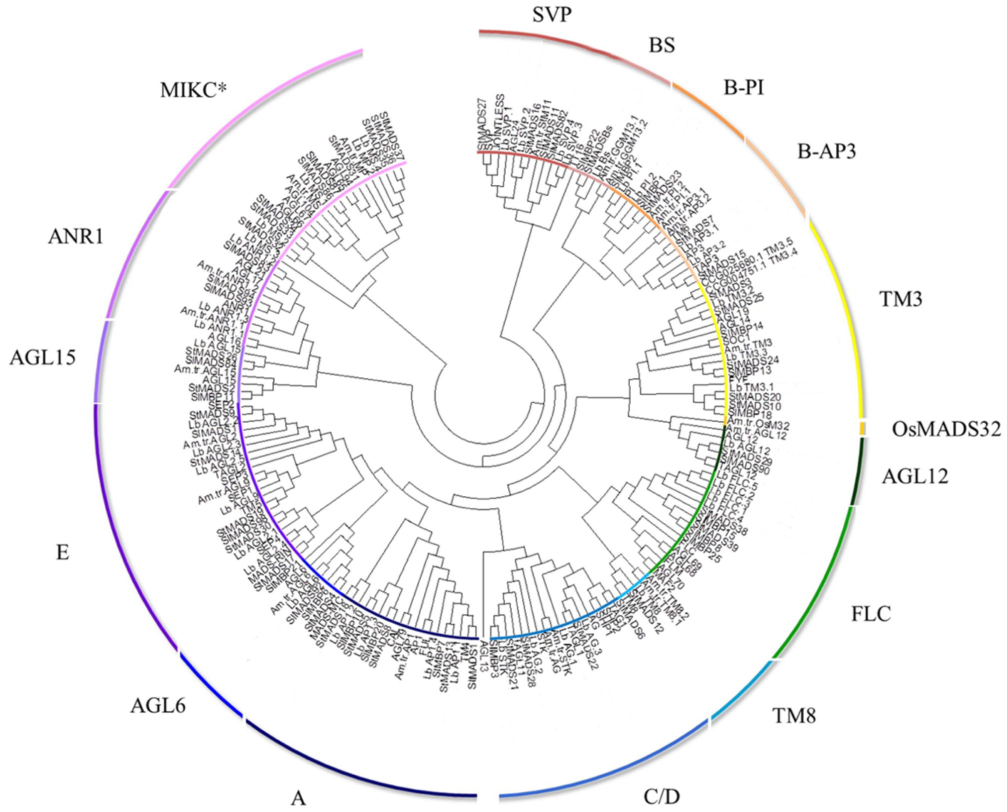

Supplementary Figure 19. Phylogenetic analysis of type II MADS-box genes from *L. barbarum*, *S. lycopersicon*, *S. tuberosum*, *A. tricopoda* and *A. thaliana*. The un-rooted neighbour-joining phylogenetic tree was constructed in MEGA5 with 1000 replicates in bootstrap values **(Supplementary Data 13).**

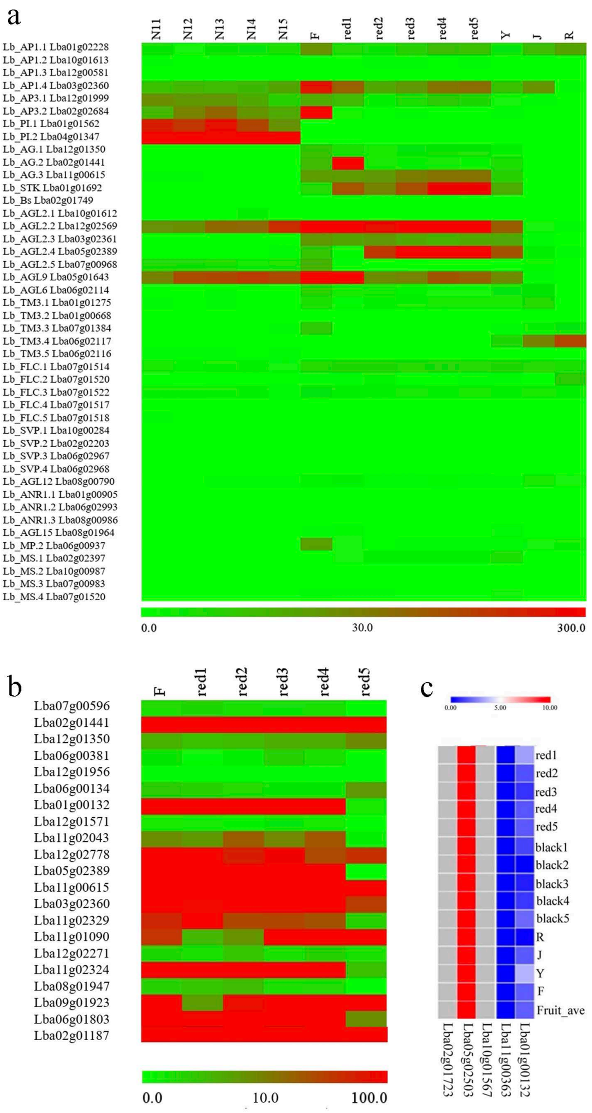

Supplementary Figure 20. The genes of expression profiles in floral and fruit development of *L. barbarum*. a. Expression profiles of type II MADS-box genes in various floral and fruit development stages and tissue. **b.** Expression profiles of fruit development- and ripening-related genes in various fruit development stages and tissue. **c.** Ripening-related NAC domain coding gene expression profiles (N11-N15, stage1-stage 5 of flower development; F: mature flower; red1-red5, stage1-stage 5 of fruit development; R, root; J, stem; Y, leaf)

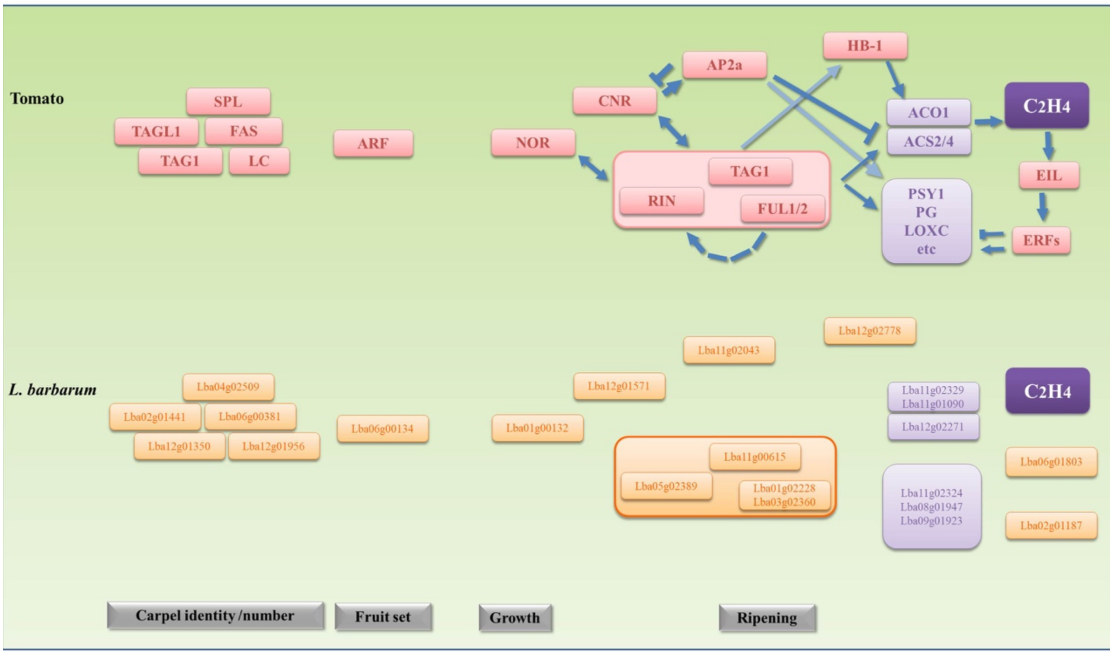

Supplementary Figure 21. Comparison of transcriptional regulators of fleshy fruit development and ripening between tomato and *L. barbarum*. Arrowheads represent positive regulatory interactions, and bar heads represent negative regulation. Light purple boxes represent a selection of affected ripening genes involved in ethylene biosynthesis (*ACO*, *ACS*) or carotenoid synthesis, softening, and flavour production. *RIN*, *TAGL*1, and *FUL*1/2 are grouped to indicate that they probably function as complexes of varying compositions. All transcription factors in *L. barbarum* are vertically aligned with their respective orthologues in tomato.

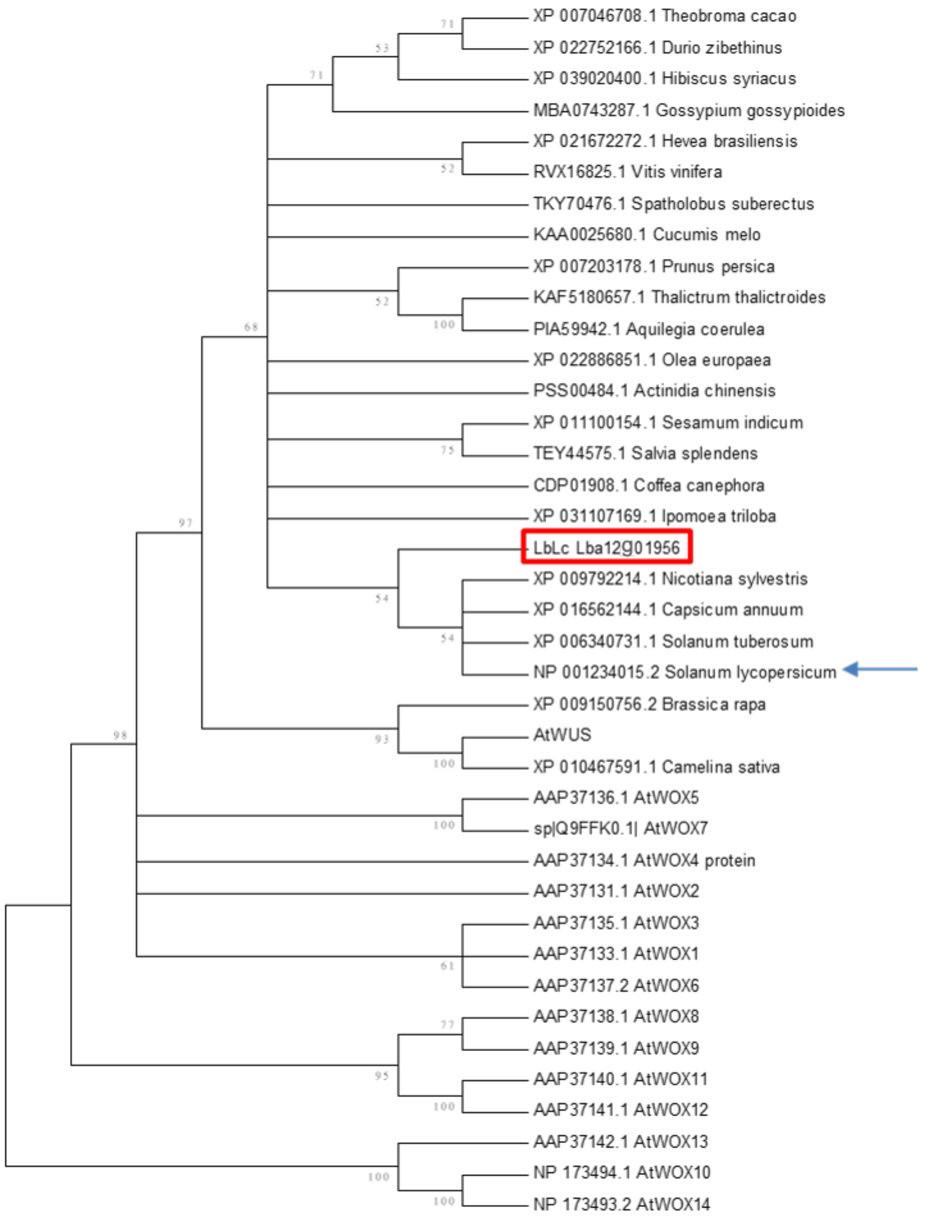

Supplementary Figure 22. Phylogenetic analysis of the *Lycium LC* gene and its homologous genes. The red rectangle denotes the *LC* gene from *Lycium*. The blue arrow denotes the *LC* gene from *Solanum lycopersicum*.

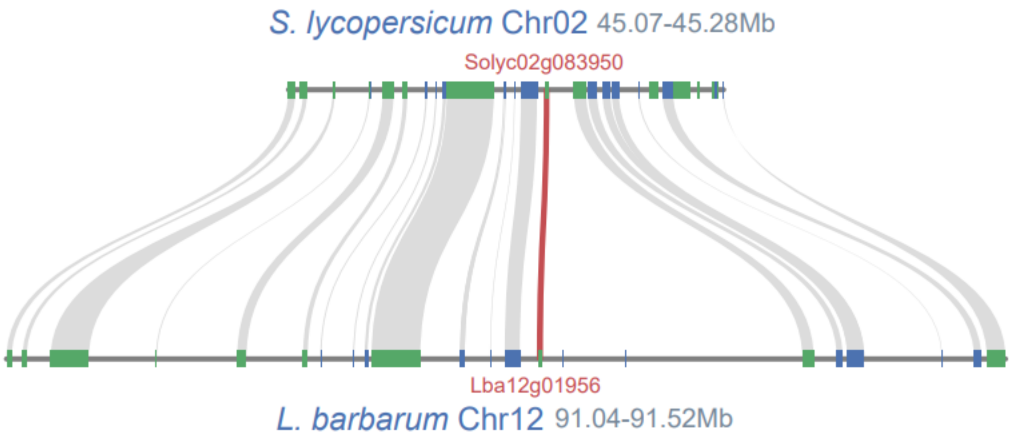

Supplementary Figure 23. Co-linear alignment of *Solanum lycopersicum* and *L. barbarum*. The red color of genes in the alignment denotes the tomato *LC* and putative *Lycium LC* genes. The grey links connect orthologues between partial regions of *S. lycopersicum* Chromosome 2 and *L. barbarum* Chromosome 12.

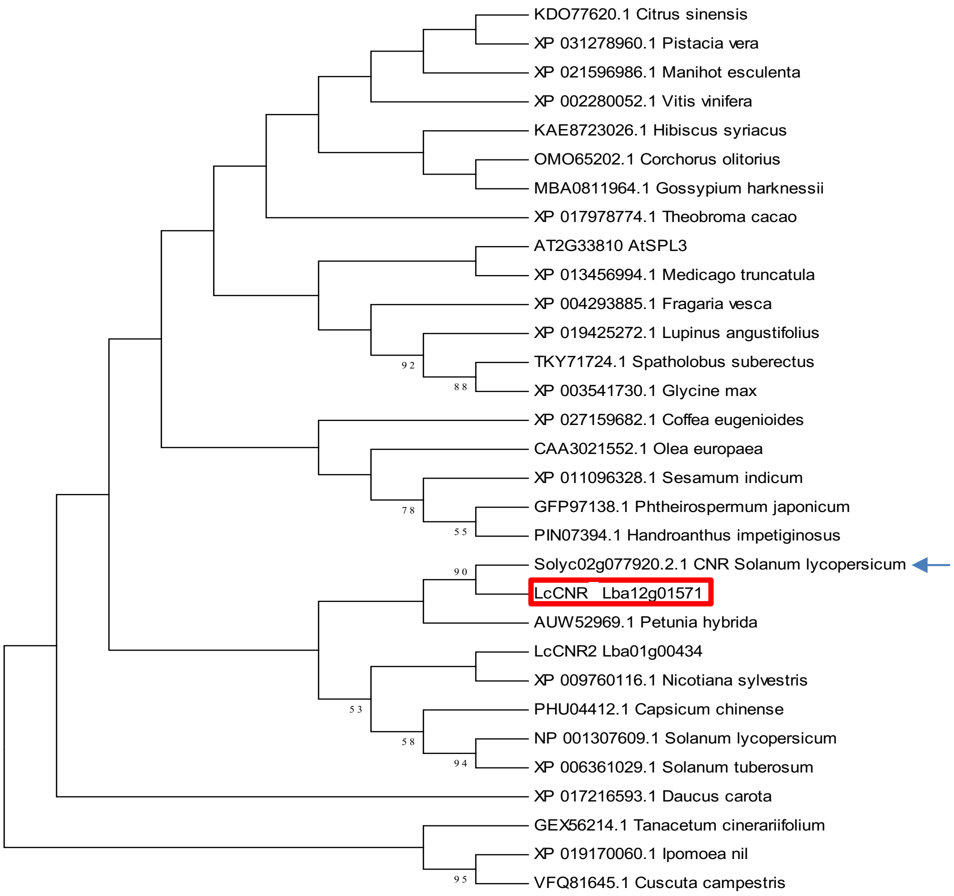

Supplementary Figure 24. Phylogenetic analysis of the *Lycium CNR* gene and its homologous genes. The red rectangle denotes the *CNR* gene from *Lycium*. The blue arrow denotes the *CNR* gene from *Solanum lycopersicum*.

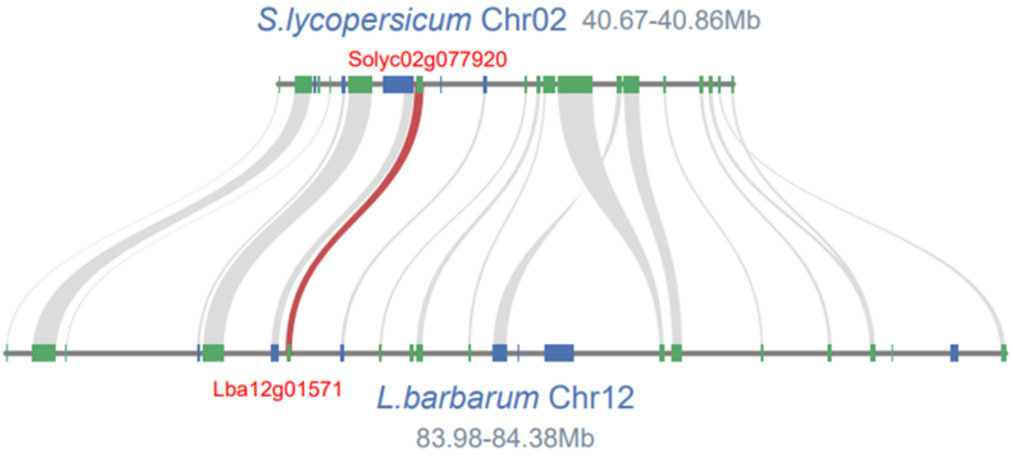

Supplementary Figure 25. Co-linear alignment of *Solanum lycopersicum* and *L. barbarum.* The red color of genes in the alignment denotes the tomato *CNR* and putative *Lycium CNR*. The grey links connect orthologues between partial regions of *S. lycopersicum* Chromosome 2 and *L. barbarum* Chromosome 12.

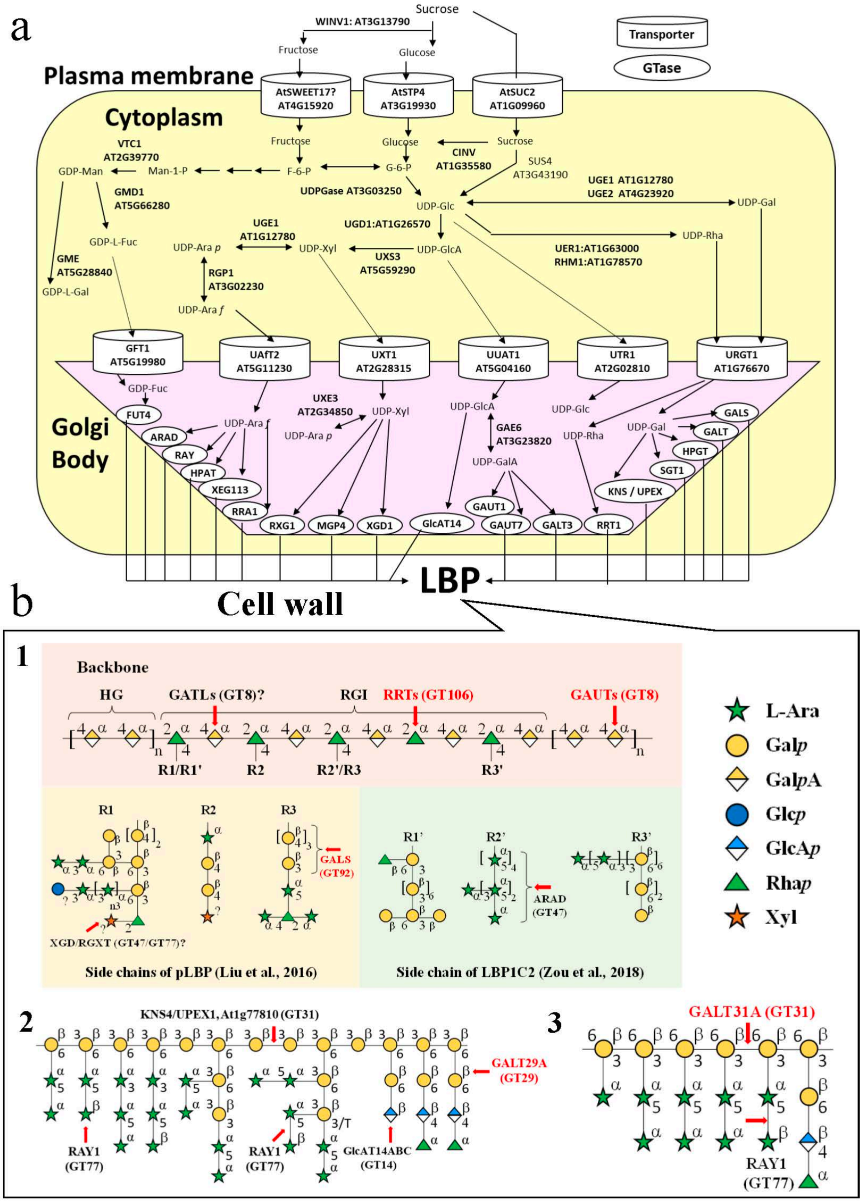

Supplementary Figure 26. Possible candidate genes for *L. barbarum* polysaccharide (LBP) biosynthesis in *Lycium* fruits. a. Proposed biosynthetic pathway of LBPs in cytoplasm and Golgi body. The biosynthetic pathway was proposed based on several *Arabidopsis* studies[6–13]. LBP biosynthesis is supposed to be generated from sucrose, mainly produced by photosynthesis in leaf tissue. LBP synthetic enzymes include Golgi-localized glycosyltransferases (also shown in this figure b) and membrane-bound sugar or NDP-sugar transporters, invertases and soluble sugar conversion-enzymes. All enzymes in this pathway are presented by *Arabidopsis* gene names and/or identifiers. The putative orthologue genes in the *Lycium* genome were identified based on BLAST searches using *Arabidopsis* sequences as queries. Only abbreviations of gene names are shown. **b.** The hypothetical structures of pectin polysaccharides (LBP) and arabinogalactan proteins (ABP) extracted from wolfberry and possible candidate GT genes for their biosynthesis. (b-1) Rhamnogalacturonan I (RG I) backbone. The model structures, proposed for pLBP[14] and

LBP1C2[15], differ in the side chains. The pLBP contains β-1,3;1,6-galactan called type II AG together with β-1,4-galactan called type I AG, while the LBP1C2 has type II AG and α-1,3;1,5-arabinan. (b-2) AGP from wolfberry has been purified using Yariv antigens and expected to have type II AGs as do AGPs from other plants[16] have been reported. (b-3) AG. β-1,6-galactan decorated with L-Ara is proposed for AG obtained as LBPA fraction[17]. The model structures of pLBP, AGP and LBPA were redrawn by Wu *et al*. 2018[18] based on the Symbol Nomenclature for Glycans (SNFG, https://www.ncbi.nlm.nih.gov/glycans/snfg.html). The model structure of LBP1C2 was redrawn from Zhou *et al*. 2018[19]. The selection of glycosyltransferase (GT) family genes shown in these figures was based on the reviews of pectin synthesis[20] and AGP synthesis[21,15] in *Arabidopsis*. The putative GT family genes in the *Lycium* genome were identified by BLAST analysis using *Arabidopsis* genes as queries. The genes with expression patterns similar to LBP accumulation during fruit developmental stages were marked by red characters.

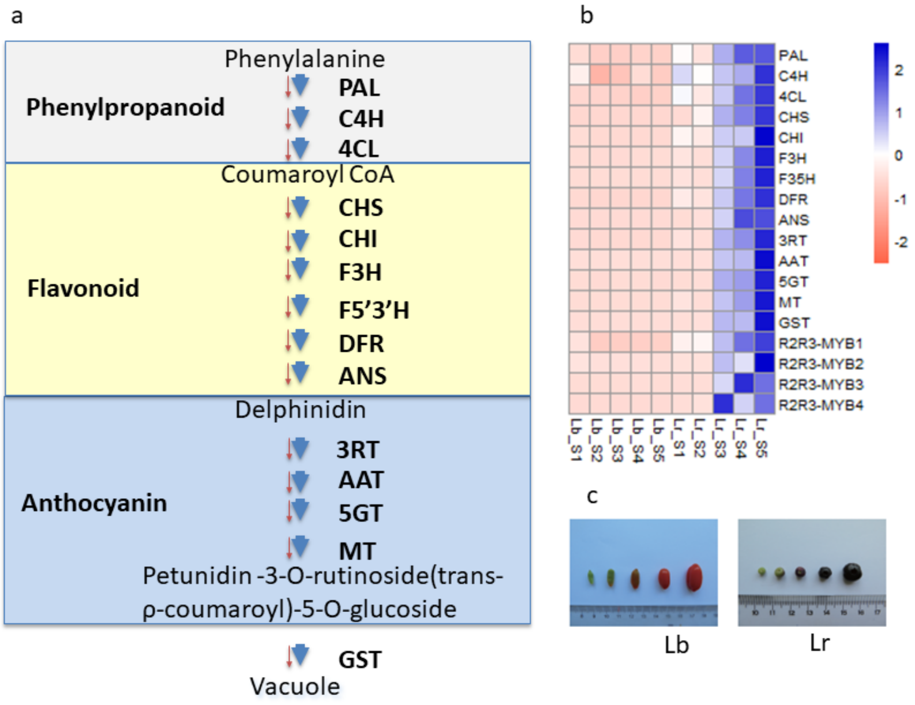

Supplementary Figure 27. **The anthocyanin biosynthesis pathway. a**. The blue arrow indicates the genes expressed for *L. ruthenium* and red arrow indicates the genes expressed for *L. barbarum*; the dashed arrow indicates the deduced pathway special for *L. ruthenium*. **b**. The heatmap of different expression levels of genes in the anthocyanin biosynthesis pathways of *L. barbarum* (Lb) and *L. ruthenium* (Lr). (PAL, phenylalanine ammonia-lyase; C4H, cinnamate 4-hydroxylase; 4CL, 4-coumaroyl-CoA-ligase; CHS, chalcone synthase; CHI, chalcone isomerase; F3H, flavanone 3-hydroxylase; F3′5′H, flavonoid 3′5′hydroxylase; DFR, dihydroflavonol 4-reductase; ANS, anthocyanidin synthase; 3GT, flavonoid 3-glucosyltransferase; 3RT, anthocyanidin 3-glucoside rhamnosyltransferase; AAT, anthocyanin acyltransferase; 5GT, flavonoid 5-glucosyltransferases; MT, anthocyanin methyltransferases;GST, glutathione S-transferase.). **c**. The developing stage of *L. barbarum* (Lb) and *L. ruthenium* (Lr) fruits.

a

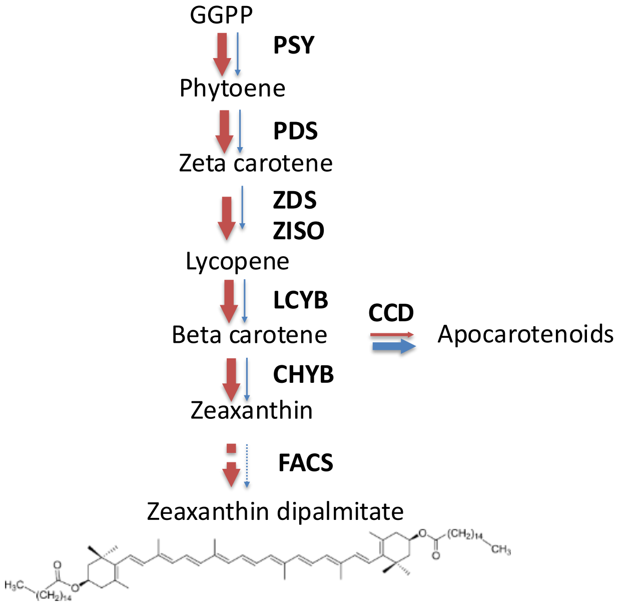

b

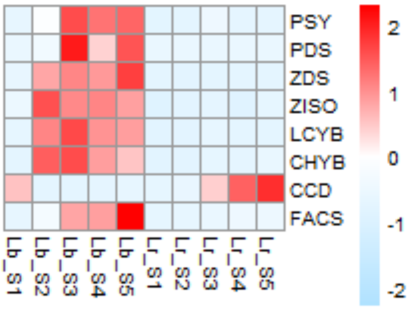

Supplementary Figure 28. The carotenoid accumulation model in *L. barbarum*. a. The carotenoid biosynthesis and accumulation pathway (The red arrow indicates the genes expressed for *L. barbarum* and blue one indicates the genes expressed for *L. ruthenium*). **b.** The heatmap of different expression levels of genes in the carotenoid biosynthesis pathways of *L. barbarum* (Lb) and *L. ruthenium* (Lr). *PSY*, phytoene synthase; *PDS*, phytoene desaturase; *ZDS*, zeta carotene desaturase; *ZISO*, zeta carotene isomerase; *LCYB*, lycopene beta cyclase; *CCD*, carotenoid cleavage dioxygenase; *CHYB*, beta carotene hydroxylase; *FACS*, fatty acyl coenzyme A synthetase.

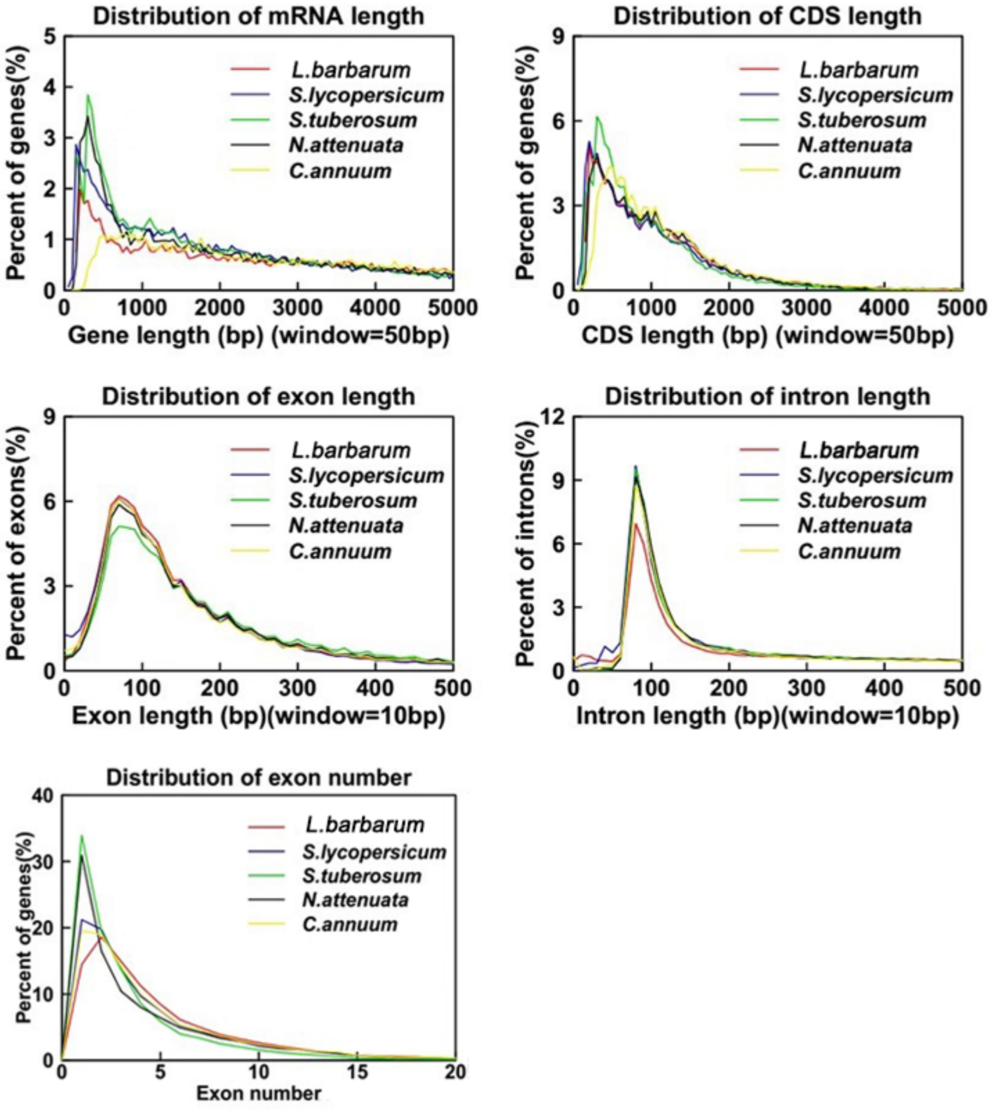

Supplementary Figure 29. Gene structure prediction results statistics. ***L. barbarum*** **compared with genetic elements of related species.** Window refers to the length represented by each point on the horizonal coordinate.

Supplementary Tables

Supplementary Table 1. Summary of sequencing of *Lycium* species.

	Sample No.	Species	Collection area	Sequencing and Assembly
1	CHG002248-P20150716-1-94_L007_1	*L. barbarum*	Ningxia, China	Chromosome-scaled assembly
2	Lr	*L. ruthenicum*	Qinghai, China	Draft assembly
3	CHG001007-P20150402-2-55_S4_L004_1	*L. chinense*	Jiangsu, China	30X coverage genome
4	CHG001008-P20150402-3-20_S1_L005_1	*L. yunnanense*	Yunnan, China	30X coverage genome
5	CHG001008-P20150402-3-57_S4_L005_1	*L. dasystemum*	Xinjiang, China	30X coverage genome
6	CHG001009-P20150402-4-35_S1_L006_1	*L. changjicum*	Xinjiang, China	30X coverage genome
7	CHG001009-P20150402-4-95_S2_L006_1	*L. cylindricum*	Xinjiang, China	30X coverage genome
8	CHG001009-P20150402-4-15_S3_L006_1	*L. truncatum*	Inner Mongolia, China	30X coverage genome
9	HE3A	*L. berlandieri*	Arizona, USA	30X coverage genome
10	HE4A	*L. andersonii*	Nevada, USA	30X coverage genome
11	HE6A	*L. depressum*	Iran	30X coverage genome
12	HE11A	*L. fremontii*	Arizona, USA	30X coverage genome
13	HE16A	*L. exsertum*	Arizona, USA	30X coverage genome

* All the voucher specimens are deposited in the Wolfberry Herbarium, and alive plants were transplanted to Goji Garden of National Wolfberry Engineering Research Center, Ningxia Academy of Agriculture and Forestry Sciences.

Supplementary Table 2. Assembly statistics of the *L. barbarum* haploid genome and *L. ruthenicum* diploid genome.

		Contig		Scaffold	
		**Size (bp)**	**Number**	**Size (bp)**	**Number**
L. barbarum	N90	3,735,486	148	3,735,486	148
	N80	5,530,307	110	5,530,307	110
	N70	6,907,604	84	6,907,604	84
	N60	8,882,014	62	8,882,014	62
	N50	10,747,313	45	10,747,313	45
	Total Length	1,669,674,389		1,669,674,389	
	Total Number(≥100bp)		405		405
	Total Number(≥2kb)		405		405
L. ruthenicum	N90	2,349	147,881	26,858	16,658
	N80	5,879	95,657	59,338	11,174
	N70	9,190	68,550	89,410	8,083
	N60	12,529	49,843	120,318	5,892
	N50	16,139	35,688	155,387	4,230
	Total Length	2,011,976,839		2,269,091,133	
	Total Number(≥100bp)		475,348		195,267
	Total Number(≥2kb)		157,334		36,912

Supplementary Table 3. Genome assembly assessment of *L. barbarum* and *L. ruthenium* by BUSCO.

Species	BUSCO	Number	Percentage
L. barbarum	Complete BUSCOs (C)	1344	97.75%
	Complete and single-copy BUSCOs (S)	1300	94.55%
	Complete and duplicated BUSCOs (D)	44	3.20%
	Fragmented BUSCOs (F)	9	0.65%
	Missing BUSCOs (M)	22	1.60%
	Total BUSCO groups searched	1,375	-
L. ruthenium	Complete BUSCOs (C)	1331	96.80%
	Complete and single-copy BUSCOs (S)	1212	88.15%
	Complete and duplicated BUSCOs (D)	119	8.65%
	Fragmented BUSCOs (F)	16	1.16%
	Missing BUSCOs (M)	28	2.04%
	Total BUSCO groups searched	1,375	-

Supplementary Table 4. The chromosome length of *L. barbarum* by Hi-C.

Chromosome ID	Length (bp)
Chr01	171,835,435
Chr02	153,603,042
Chr03	149,360,239
Chr04	149,041,705
Chr05	143,530,904
Chr06	137,364,012
Chr07	134,699,017
Chr08	133,540,852
Chr09	126,384,085
Chr010	125,083,490
Chr011	124,265,054
Chr012	106,533,526

Supplementary Table 5. Assembly statistics of the *L. barbarum* genome by Hi-C.

	Contig		Scaffold	
	**Length**	**Number**	**Length**	**Number**
N50	9,356,405	51	137,364,012	6
N90	3,112,385	167	124,265,054	11
Total	1,655,078,361	-	1,655,241,361	-

Supplementary Table 6. Prediction of gene structures of the *L. barbarum* and *L. ruthenicum* genome.

Species		Gene set	Number	Average mRNA length(bp)	Average CDS length(bp)	Average exon per gene	Average exon length(bp)	Average intron length(bp)
L. barbarum	Denovo	Augustus	36,556	5707.85	1141.18	4.93	231.57	1162.63
		SNAP	77,901	6370.48	679.26	4.29	158.30	1729.35
	Homolog	*S. melongena*	65,366	3305.59	838.80	3.25	258.09	1096.33
		S. lycopersicum	118,235	3246.44	730.72	2.87	254.33	1343.08
		S. tuberosum	108,782	3026.06	684.19	2.43	281.48	1636.91
		C. annuum	97,408	4049.69	895.09	3.15	284.45	1469.52
		N. attenuata	91,253	3497.82	817.72	3.09	264.82	1283.68
		P. inflata	131,303	3733.00	777.62	2.98	260.92	1492.40
		Transctriptome	110,914	4453.04	1581.56	3.46	456.64	1165.61
		Maker	33,581	5839.83	1122.67	5.23	214.73	1115.61
L. ruthenicum	Denovo	fgensh	67,120	2,587	823	4.3	192	538
		Augustus	103,965	4,296	798	4.2	192	1,108
		GlimmerHMM	106,629	1,229	547	2.4	228	488
	Homolog	*S. lycopersicum*	123,545	1,705	640	2.4	266	757
		S. melongena	263,269	890	369	1.6	227	837
		S. tuberosum	186,895	1,144	520	1.9	277	712
		RNA-seq	55,888	4,183	1,539	3.8	402	935
		Combine-filter	32,711	4,040	1,138	4.5	255	746

Supplementary Table 7. BUSCO assessment of *L. barbarum* and *L. ruthenicum* genome annotation.

Type	BUSCO	Number	Percentage
L. barbarum	Complete BUSCO (C)	1281	93.16%
	Complete and single-copy BUSCOs (S)	1251	90.98%
	Complete and duplicated BUSCOs (D)	30	2.18%
	Fragmented BUSCOs (F)	36	2.62%
	Missing BUSCOs (M)	58	4.22%
	Total BUSCO groups searched	1,375	-
L. ruthenicum	Complete BUSCOs (C)	1229	89.38%
	Complete and single-copy BUSCOs (S)	1143	83.13%
	Complete and duplicated BUSCOs (D)	86	6.25%
	Fragmented BUSCOs (F)	60	4.36%
	Missing BUSCOs (M)	86	6.25%
	Total BUSCO groups searched	1,375	-

Supplementary Table 8. Non-coding RNA annotation results of *L. barbarum* and *L. ruthenicum* genome.

	Type		Copy	Average length (bp)	Total length (bp)	% of genome
L. barbarum	miRNA		151	126.98	19,174	0.0011
	tRNA		1,255	75.21	94,393	0.0057
	rRNA	rRNA	2,361	115.7	273,178	0.016
		18S	157	369.11	57,950	0.0035
		28S	116	125.82	14,595	0.00087
		5.8S	40	128.15	5,126	0.00031
		5S	2,048	95.46	195,507	0.0117
	snRNA	SnRNA	1,243	116.94	145,353	0.0087
		CD-box	724	107.64	77,928	0.0047
		HACA-box	77	122.92	9,465	0.00057
		Splicing	442	131.13	557,960	0.0035
L. ruthenicum	miRNA		165	126.62	20,892	0.000921
	tRNA		1,602	75.29	120,607	0.005315
	rRNA	rRNA	1,728	101.1	174,695	0.007699
		18S	194	188.66	36,600	0.001613
		28S	60	96	5,760	0.000254
		5.8S	31	109.1	3,382	0.000149
		5S	1,443	89.36	128,953	0.005683
	snRNA	snRNA	1,736	113.25	196,607	0.008665
		CD-box	1,112	104.57	116,281	0.005125
		HACA-box	93	122.72	11,413	0.000503
		splicing	531	129.78	68,913	0.003037

Supplementary Table 9. The genes involved in anthocyanin biosynthesis pathway.

Name	Gene ID
PAL	Lba03g00285
C4H	Lba01g02920
4CL	Lba03g01775
CHS	Lba05g00342
CHI	Lba05g00431
F3H	Lba12g01948
F3'5'H	Lba06g00197
DFR	Lba12g02047
ANS	Lba04g00366
3RT	Lba04g00379
AAT	Lba07g01581
5GT	Lba07g01991
MT	Lba11g01977
GST	Lba12g00873

Supplementary Table 10. Gene expression levels (FPKMs) of anthocyanin biosynthesis-related in 5 stages along the *Lycium* fruit ripening.

Gene_Name	Gene_ID	Lb_S1	Lb_S2	Lb_S3	Lb-S4	Lb_S5	Lr_S1	Lr_S2	Lr_S3	Lr_S4	Lr_S5
**PAL**	Lba03g00285	25.43	2.48	2.06	7.66	3.52	86.16	35.72	188.43	284.65	289.94
**C4H**	Lba01g02920	343.4	64.38	137.84	254.42	178.06	535.39	409.32	585.26	661.81	1023.33
**4CL**	Lba03g01775	22.32	16.47	8.94	5.59	7.03	131.52	63.66	200.09	328.69	418.85
**CHS**	Lba05g00342	4.83	0.15	1.73	0.59	0.36	54.09	375.66	1182.49	1575.29	2164.34
**CHI**	Lba05g00431	85.98	2.8	1.93	6.68	5.06	804.82	483.69	2033.21	2070.82	5648.4
**F3H**	Lba12g01948	154.11	40.28	81.62	136.23	137.28	786.94	354.98	4458.49	7784.22	12388.5
**F3'5'H**	Lba06g00197	26.23	0.99	0.89	0.07	0.41	237.39	83.71	2110.89	3973.19	6036.25
**DFR**	Lba12g02047	46.63	0.79	0.53	0.09	0	502.64	313.25	1830.89	3157.6	4253.71
**ANS**	Lba04g00366	13.12	1.33	1.94	0.92	2.09	39.74	130.51	1533.41	3493.35	3457.02
**3RT**	Lba04g00379	22.54	0.08	0.69	1.7	1.22	186.5	95.52	1675.98	2147.02	3426.64
**AAT**	Lba07g01581	0	0	0	0	0	3.1	32.27	2236.52	2468.69	6006.14
**5GT**	Lba07g01991	1.95	0.01	0.09	0.09	0.06	3.45	18.75	720.86	815.71	1451.23
**MT**	Lba11g01977	2.05	0	0.18	0	0	466.85	92.41	4609	6079.09	11494.1
**GST**	Lba12g00873	0	0	0	0	0	19.86	56.16	1871.71	1845.03	4438.43
**R2R3-MYB1**	Lba04g02548	48.65	1.2	2.25	7.67	8.12	82.66	96.16	210.75	322.15	390.97
**R2R3-MYB2**	Lba05g02024	5	1.2	0.71	0.66	0	2.05	2.47	78.3	56.27	205.94
**R2R3-MYB3**	Lba08g01154	0	0	0.99	2.65	1.52	5.56	4.5	168.2	471.59	350.12
**R2R3-MYB4**	Lba05g02025	0	0	0	0	0	1.46	4.79	138.79	53.97	104.21

Supplementary Table 11. Gene expression levels (FPKMs) of carotenoid biosynthesis-related in 5 stages along the *Lycium* fruit ripening.

Gene_Name	Gene_ID	Lb_S1	Lb_S2	Lb_S3	Lb-S4	Lb_S5	Lr_S1	Lr_S2	Lr_S3	Lr_S4	Lr_S5
**PSY**	Lba11g02324	69.17	309.20	973.30	833.29	882.34	24.08	34.43	149.13	42.28	48.57
**PDS**	Lba03g03127	0.00	35.63	436.00	164.31	350.32	0.00	0.00	0.00	0.00	0.00
**ZDS**	Lba06g01695	122.72	841.08	984.92	898.58	1302.08	56.67	46.57	66.86	71.07	65.22
**ZISO**	Lba07g02021	65.95	325.45	262.22	266.94	236.66	18.72	33.43	40.57	23.79	44.27
**LCYB**	Lba05g00383	58.71	821.39	1054.14	767.51	728.41	5.32	16.78	68.90	27.75	36.51
**CHYB**	Lba03g01505	144.39	5697.26	6088.64	4238.63	3368.92	17.60	83.47	335.50	269.60	524.63
**CCD**	Lba03g01270	406.02	17.27	18.48	27.79	40.91	36.14	53.35	371.58	666.75	801.72
**FACS**	Lba07g02085	28.66	233.73	843.16	870.82	1659.95	14.81	26.74	78.15	131.57	125.94

Supplementary Table 12. Dating of whole genome triplication event.

	Lower CI of Divtime with *Lycium* (Mya)	Divtime with *Lycium* (Mya)	Upper CI of Divtime with *Lycium* (Mya)	Divide log_{10} *Ks*	Divide *Ks*	Upper CI λ	λ	lower CI λ	WGT log10 *Ks*	WGT *Ks*	WGT time (Mya)	95% CI of WGT time
PAXI	40	40.47	41	-0.46	0.34	0.0085	0.008401	0.008293	-0.21	0.62	73.80	72.94-74.96
PINF	40	40.47	41	-0.46	0.34	0.0085	0.008401	0.008293	-0.23	0.59	70.23	69.41-71.15
NTAB	29.53	34.17	37.47	-0.52	0.3	0.01015916	0.00878	0.008006	-0.22	0.6	68.34	59.06-74.94
CANN	21.06	25.93	30.84	-0.64	0.23	0.010921178	0.00887	0.007458	-0.17	0.67	75.54	61.35-89.84
SMEL	21.06	25.93	30.84	-0.55	0.28	0.013295347	0.010798	0.009079	-0.15	0.7	64.83	52.65-77.10
SLYC	21.06	25.93	30.84	-0.6	0.25	0.011870845	0.009641	0.008106	-0.17	0.67	69.49	56.44-82.65
STUB	21.06	25.93	30.84	-0.62	0.24	0.011396011	0.009256	0.007782	-0.21	0.62	66.99	54.41-79.67

Supplementary References

1. Li, L., Stoeckert, Jr., C. J. & Roos, D. S. OrthoMCL: Identification of Ortholog Groups for Eukaryotic Genomes. *Genome Res*. **13**, 2178–2189 (2003).
2. Guindon, S. *et al*. New algorithms and methods to estimate maximum likelihood phylogenies: assessing the performance of PhyML 3.0. *Syst. Biol.* **59**, 307–321 (2010).
3. Yang, Z. PAML 4: phylogenetic analysis by maximum likelihood. *Mol. Biol. Evol*. **24**, 1586–1591 (2007).
4. Kumar, S., Stecher, G., Suleski, M. and Hedges, S. B. TimeTree: a resource for timelines, timetrees, and divergence times. *Mol. Biol. Evol*. **34**,1812–1819 (2017).
5. Miller, J. S., Kostyun, J. L. Functional gametophytic self-incompatibility in a peripheral population of *Solanum peruvianum* (Solanaceae). *Heredity* **107**, 30–39 (2011).
6. Truernit, E., Schmid, J., Epple, P., Illig, J. & Sauer, N. The sink-specific and stress-regulated *Arabidopsis STP4* gene: enhanced expression of a gene encoding a monosaccharide transporter by wounding, elicitors, and pathogen challenge. *Plant Cell* **8,** 2169–2182 (1996).
7. Chandran, D., Reinders, A. & Ward, J. M. Substrate specificity of the *Arabidopsis thaliana* sucrose transporter AtSUC2. *J. Biol. Chem.* **278**, 44320–44325 (2003).
8. Sherson, S. M. *et al*. Roles of cell-wall invertases and monosaccharide transporters in the growth and development of *Arabidopsis*. *J. Exp. Bot.* **54**, 525–531 (2003).
9. Meng, M., Wilczynska, M. & Kleczkowski, L. A. Molecular and kinetic characterization of two UDP-glucose pyrophosphorylases, products of distinct genes, from *Arabidopsis*. *Biochim. Biophys. Acta.* **1784**, 967–972 (2008).
10. Barratt, D. P. *et al*. Normal growth of *Arabidopsis* requires cytosolic invertase but not sucrose synthase. *Proc. Natl. Acad. Sci. U. S. A.* **106**, 13124–13129 (2009).
11. Handford, M. *et al*. *Arabidopsis thaliana AtUTr7* encodes a Golgi-localized UDP–Glucose/UDP–Galactose transporter that affects lateral root emergence. *Mol. Plant* **5,** 1263–1280 (2012).
12. Guo, W. J. *et al*. a facilitative transporter, mediates fructose transport across the tonoplast of *Arabidopsis* roots and leaves. *Plant Physiol.* **164**, 777–789 (2014).
13. Sechet, J. *et al*. Suppression of *Arabidopsis* GGLT 1 affects growth by reducing the L-galactose content and borate cross-linking of rhamnogalacturonan-II. *Plant J.* **96**, 1036–1050 (2018).
14. Zong, Y. *et al.* Functional MYB transcription factor encoding gene AN2 is associated with anthocyanin biosynthesis in *Lycium ruthenicum* Murray. *BMC Plant Biol.* **19**, 169 (2019).
15. Morrone, L. A. *et al*. Natural compounds and retinal ganglion cell neuroprotection. *Prog. Brain Res.* **220,** 257–281 (2015).

16. Redgwell, R. J. *et al*. Cell wall polysaccharides of Chinese Wolfberry (*Lycium barbarum*): Part 2. Characterisation of arabinogalactan-proteins. *Carbohydr. Polym.* **84**, 1075–1083 (2011).
17. Yuan, Y. *et al.* Structure identification of a polysaccharide purified from *Lycium barbarum* fruit. *Int. J. Biol. Macromol.* **82**, 696–701 (2016).
18. Wu, D. T. *et al*. Review of the structural characterization, quality evaluation, and industrial application of *Lycium barbarum* polysaccharides. *Trends Food Sci. Technol.* **79**, 171–183 (2018).
19. Zhou, L. *et al*. A pectin from fruits of *Lycium barbarum* L. decreases β-amyloid peptide production through modulating APP processing. *Carbohydr. Polym.* **201,** 65–74 (2018).
20. Anderson C.T. Pectic Polysaccharides in Plants: Structure, Biosynthesis, Functions, and Applications. In: Cohen E., Merzendorfer H. (eds). Extracellular Sugar-Based Biopolymers Matrices. *Biologically-Inspired Systems*, pp. 487–514 (Springer, Cham, 2019).
21. Showalter, A. M. and Basu, D. Extensin and Arabinogalactan-Protein Biosynthesis: Glycosyltransferases, Research Challenges, and Biosensors. *Front. Plant Sci*. **7**, 814 (2016).
