# Supplementary material for: Wolfberry genomes and the evolution of Lycium (Solanaceae)
Source: Commun Biol. 2021 Jun 3;4:671. doi: 10.1038/s42003-021-02152-8 (PMC8175696; doi:10.1038/s42003-021-02152-8)
Supplement: Supplementary file 3 — Description of Additional Supplementary Files [file 42003_2021_2152_MOESM3_ESM.pdf]

Description of Additional Supplementary Files

File name: Supplementary Data 1

Description: Gene Ontology (GO) enrichment result of Solanaceae's gene families.

File name: Supplementary Data 2

Description: The significant expansion and contraction genes families of all genera in Solanaceae.

File name: Supplementary Data 3

Description: Gene Ontology (GO) enrichment result of Solanaceae's expanded gene families.

File name: Supplementary Data 4

Description: KEGG enrichment result of Solanaceae's expanded gene families.

File name: Supplementary Data 5

Description: Gene Ontology (GO) enrichment result of unique genes of L. barbarum.

File name: Supplementary Data 6

Description: Gene Ontology (GO) enrichment result of expanded gene families of L. barbarum.

File name: Supplementary Data 7

Description: KEGG enrichment result of expanded gene families of L. barbarum.

File name: Supplementary Data 8

Description: Supplementary Data 8. The basic information of S-RNases related sequences found in Solanaceae genomes, and from species with S-RNase based SI, maize and barley.

File name: Supplementary Data 9

Description: The RNases-T2 genes, their physical positions, and the information whether located in hybridization hotspots.

File name: Supplementary Data 10

Description: The putative cellulose synthetic genes in the Lycium genome.

File name: Supplementary Data 11

Description: The putative lignin synthetic genes in the Lycium genome.

File name: Supplementary Data 12

Description: The expression levels of the tandem duplicated genes of CAD and LAC in the stem transcriptomes.

File name: Supplementary Data 13

Description: List of 87 MADS-box genes identified in L. barbarum.

File name: Supplementary Data 14

Description: Putative genes for LBP biosynthesis in the Lycium genome and their expression levels.

File name: Supplementary Data 15

Description: Number of putative pectin biosynthetic genes in Lycium and other Solanaceae genomes.

File name: Supplementary Data 16

Description: Genes involved in biosynthesis of nucleotide sugars that supply pectin biosynthesis.

File name: Supplementary Data 17

Description: Numbers of genes involved in biosynthesis of nucleotide sugars that supply pectin biosynthesis in Lycium and other Solanaceae genomes.

File name: Supplementary Data 18

Description: Source data for Figure 1

File name: Supplementary Data 19

Description: Source data for Figure 2

File name: Supplementary Data 20

Description: Source data for Figure 4
