# Supplementary material for: Wolfberry genomes and the evolution of Lycium (Solanaceae)
Source: Commun Biol. 2021 Jun 3;4:671. doi: 10.1038/s42003-021-02152-8 (PMC8175696; doi:10.1038/s42003-021-02152-8)
Supplement: Supplementary file 24 — Reporting Summary [file 42003_2021_2152_MOESM24_ESM.pdf]

nature research

Corresponding author(s): Zhong-Jian Liu

Last updated by author(s): Apr 1, 2021

# Reporting Summary

Nature Research wishes to improve the reproducibility of the work that we publish. This form provides structure for consistency and transparency in reporting. For further information on Nature Research policies, see our Editorial Policies and the Editorial Policy Checklist.

## Statistics

For all statistical analyses, confirm that the following items are present in the figure legend, table legend, main text, or Methods section.

| n/a | Confirmed | |
|---|---|---|
| ☐ | ☒ | The exact sample size (*n*) for each experimental group/condition, given as a discrete number and unit of measurement |
| ☐ | ☒ | A statement on whether measurements were taken from distinct samples or whether the same sample was measured repeatedly |
| ☒ | ☐ | The statistical test(s) used AND whether they are one- or two-sided<br>*Only common tests should be described solely by name; describe more complex techniques in the Methods section.* |
| ☒ | ☐ | A description of all covariates tested |
| ☒ | ☐ | A description of any assumptions or corrections, such as tests of normality and adjustment for multiple comparisons |
| ☒ | ☐ | A full description of the statistical parameters including central tendency (e.g. means) or other basic estimates (e.g. regression coefficient) AND variation (e.g. standard deviation) or associated estimates of uncertainty (e.g. confidence intervals) |
| ☒ | ☐ | For null hypothesis testing, the test statistic (e.g. *F*, *t*, *r*) with confidence intervals, effect sizes, degrees of freedom and *P* value noted<br>*Give P values as exact values whenever suitable.* |
| ☐ | ☒ | For Bayesian analysis, information on the choice of priors and Markov chain Monte Carlo settings |
| ☒ | ☐ | For hierarchical and complex designs, identification of the appropriate level for tests and full reporting of outcomes |
| ☒ | ☐ | Estimates of effect sizes (e.g. Cohen's *d*, Pearson's *r*), indicating how they were calculated |

*Our web collection on statistics for biologists contains articles on many of the points above.*

## Software and code

Policy information about availability of computer code

| | |
|---|---|
| Data collection | Leaves from a haploid seedling of Lycium barbarum were picked for DNA extraction, and were used for de novo sequencing and assembly. DNA was extracted and a 20 kb insert library was constructed according to the PacBio RSII protocol and sequenced subsequently on the PacBio platform. For Lycium ruthenicum, DNA was extracted from the leaves, and libraries with insert sizes of 200 bp, 500 bp, 800 bp, 2,000 bp, 5,000 bp and 10,000 bp were constructed for de novo sequencing on the Hiseq 4000 platform. A total of 13 wolfberry accessions from all over China were collected, and DNA from single plants was extracted as described above. Paired-end libraries with insert sizes of 500 bp were constructed with the same protocol as de novo sequencing and sequenced on the Hiseq 4000 platform. |
| Data analysis | For L. barbarum, smartdenovo was used to assemble the genome, and SOAPdenovo2 was used for L. ruthenicum. Reads of the 13 resequencing were mapped with the BWA-MEM version using the default parameters 0.7.8. The obtained alignments were filtered using SAMtools version 0.1.19. Duplicated reads were marked using MarkDuplicated from Picard tools version 1.119. Finally, the SNPs for each genotype across the 13 accessions were identified using Genome Analysis Toolkit (GATK) version 2.4.9. |

For manuscripts utilizing custom algorithms or software that are central to the research but not yet described in published literature, software must be made available to editors and reviewers. We strongly encourage code deposition in a community repository (e.g. GitHub). See the Nature Research guidelines for submitting code & software for further information.

## Data

Policy information about availability of data

All manuscripts must include a data availability statement. This statement should provide the following information, where applicable:

- Accession codes, unique identifiers, or web links for publicly available datasets
- A list of figures that have associated raw data
- A description of any restrictions on data availability

Genome sequences and whole-genome assemblies have been submitted to the National Center for Biotechnology Information (NCBI) database under PRJNA640228

# Field-specific reporting

Please select the one below that is the best fit for your research. If you are not sure, read the appropriate sections before making your selection.

☒ Life sciences ☐ Behavioural & social sciences ☐ Ecological, evolutionary & environmental sciences

For a reference copy of the document with all sections, see nature.com/documents/nr-reporting-summary-flat.pdf

# Life sciences study design

All studies must disclose on these points even when the disclosure is negative.

| | |
|---|---|
| Sample size | We produced genome data for Lycium barbarum and Lycium ruthenicum, together with 13 wolfberry accessions' resequencing data from all over China. |
| Data exclusions | No data exclusions in this manuscript. |
| Replication | No replication in this manuscipt. |
| Randomization | No randomization in this manuscript as samples were not allocated into experimental groups. |
| Blinding | No blinding in this manuscript as the data were not allocated into groups |

# Reporting for specific materials, systems and methods

We require information from authors about some types of materials, experimental systems and methods used in many studies. Here, indicate whether each material, system or method listed is relevant to your study. If you are not sure if a list item applies to your research, read the appropriate section before selecting a response.

## Materials & experimental systems

| n/a | Involved in the study | |
|---|---|---|
| ☒ | ☐ | Antibodies |
| ☒ | ☐ | Eukaryotic cell lines |
| ☒ | ☐ | Palaeontology and archaeology |
| ☒ | ☐ | Animals and other organisms |
| ☒ | ☐ | Human research participants |
| ☒ | ☐ | Clinical data |
| ☒ | ☐ | Dual use research of concern |

## Methods

| n/a | Involved in the study | |
|---|---|---|
| ☒ | ☐ | ChIP-seq |
| ☒ | ☐ | Flow cytometry |
| ☒ | ☐ | MRI-based neuroimaging |
